# Comparative genomics uncovers the evolutionary history, demography, and molecular adaptations of South American canids

**DOI:** 10.1073/pnas.2205986119

**Published:** 2022-08-15

**Authors:** Daniel E. Chavez, Ilan Gronau, Taylor Hains, Rebecca B. Dikow, Paul B. Frandsen, Henrique V. Figueiró, Fabrício S. Garcez, Ligia Tchaicka, Rogério C. de Paula, Flávio H. G. Rodrigues, Rodrigo S. P. Jorge, Edson S. Lima, Nucharin Songsasen, Warren E. Johnson, Eduardo Eizirik, Klaus-Peter Koepfli, Robert K. Wayne

**Affiliations:** ^a^Department of Ecology and Evolutionary Biology, University of California, Los Angeles, CA 90095;; ^b^Biodesign Institute, School of Life Sciences, Arizona State University, Tempe, AZ 85287;; ^c^Efi Arazi School of Computer Science, Reichman University, Herzliya 46150, Israel;; ^d^Committee on Evolutionary Biology, University of Chicago, Chicago, IL 60637;; ^e^Data Science Lab, Office of the Chief Information Officer, Smithsonian Institution, Washington, DC 20560;; ^f^Department of Plant and Wildlife Sciences, Brigham Young University, Provo, UT 84602;; ^g^Smithsonian’s National Zoo and Conservation Biology Institute, Center for Species Survival, Front Royal, VA 22630;; ^h^School of Health and Life Sciences, Pontifical Catholic University of Rio Grande do Sul, Porto Alegre, 90619-900, Brazil;; ^i^Rede de Biodiversidade e Biotecnologia da Amazônia, Curso de Pós-Graduação em Recursos Aquáticos e Pesca, Universidade Estadual do Maranhão, São Luis, 2016-8100, Brazil;; ^j^Centro Nacional de Pesquisa e Conservação de Mamíferos Carnívoros, Instituto Chico Mendes de Conservação da Biodiversidade, 12952-011, Atibaia, Brazil;; ^k^Department of Genetics, Ecology and Evolution, Universidade Federal de Minas Gerais, Belo Horizonte, 31270-901, Brazil;; ^l^Centro Nacional de Avaliação da Biodiversidade e de Pesquisa e Conservação do Cerrado, Instituto Chico Mendes de Conservação da Biodiversidade, Brasilia, 70670-350, Brazil;; ^m^Private address, Nova Xavantina, MT, 78690-000, Brazil;; ^n^Instituto Pró-Carnívoros, Atibaia, 12945-010, Brazil;; ^o^Instituto Nacional de Ciência e Tecnologia em Ecologia Evolução Conservação da Biodiverside, Universidade Federal de GoiásGoiânia, 74690-900, Brazil;; ^p^Smithsonian-Mason School of Conservation, George Mason University, Front Royal, VA 22630

**Keywords:** South America, genomes, Canidae, neotropics, positive selection

## Abstract

The remarkable radiation of South American (SA) canids produced 10 extant species distributed across diverse habitats, including disparate forms such as the short-legged, hypercarnivorous bush dog and the long-legged, largely frugivorous maned wolf. Despite considerable research spanning nearly two centuries, many aspects of their evolutionary history remain unknown. Here, we analyzed 31 whole genomes encompassing all extant SA canid species to assess phylogenetic relationships, interspecific hybridization, historical demography, current genetic diversity, and the molecular bases of adaptations in the bush dog and maned wolf. We found that SA canids originated from a single ancestor that colonized South America 3.9 to 3.5 Mya, followed by diversification east of the Andes and then a single colonization event and radiation of *Lycalopex* species west of the Andes. We detected extensive historical gene flow between recently diverged lineages and observed distinct patterns of genomic diversity and demographic history in SA canids, likely induced by past climatic cycles compounded by human-induced population declines. Genome-wide scans of selection showed that disparate limb proportions in the bush dog and maned wolf may derive from mutations in genes regulating chondrocyte proliferation and enlargement. Further, frugivory in the maned wolf may have been enabled by variants in genes associated with energy intake from short-chain fatty acids. In contrast, unique genetic variants detected in the bush dog may underlie interdigital webbing and dental adaptations for hypercarnivory. Our analyses shed light on the evolution of a unique carnivoran radiation and how it was shaped by South American topography and climate change.

## Significance

The diversification of canids in South America represents one of the most striking radiations of carnivorous mammals, comprising both small and large species, as well as hypercarnivorous and frugivorous forms. However, the timing, relationships, and geographic and climatic constraints on this radiation are not well understood. We show that canids colonized that continent from a single ancestral species between 3.9 and 3.5 million years ago. Canids first diversified in eastern South America, followed by a colonization and diversification west of the Andes with demographic histories influenced by habitat shifts during Pleistocene climatic cycles. We show that the phenotypic divergence of the bush dog and maned wolf reflect changes in the regulation and composition of genes underlying dental and skeletal traits.

**Fig. 1.**
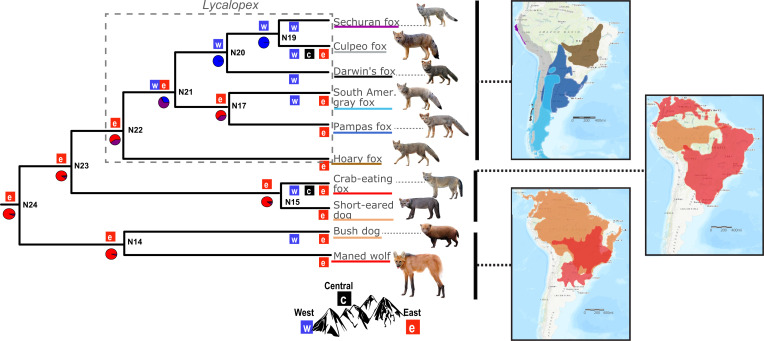
Species tree and ancestral area reconstruction of SA canids. The species tree was estimated by applying ASTRAL-III (45) to 6,716 genomic windows (25 kb each). A total of 31 genomes were included in the analysis (*SI Appendix*, Table S1), but only the clade containing SA canids is shown here (see complete tree in *SI Appendix*, Fig. S1). All nodes had 100% bootstrap support based on 100 replicates. The best-fitting BioGeoBEARS (46) model was DIVALIKE (dispersal vicariance) with the parameter “J” that represents a founder event (*SI Appendix*, Fig. S2 and Table S2). Colored boxes on each node indicate estimated ancestral ranges, while boxes at terminal branches indicate current species distribution (“e,” east of the Andes; “c,” central region of the Andes; and “w,” west of the Andes). The probabilities of these ancestral regions are shown in the pie charts below each node. Purple-colored pie charts indicate a distribution on the west and east of the Andes. The maps on the *Right* represent the distribution of species within three major clades. The colored distributions on the map match the colors underlining species names. Canid illustrations from ref. 2 are used with permission from Princeton University Press.

The arrival and diversification of mammals in South America during the 66-My history of the Cenozoic has long interested paleontologists and evolutionary biologists (1). The immigration of carnivorous mammals such as canids, felids, procyonids, and ursids from North America into South America during the Great American Biotic Interchange in the Pliocene was especially noteworthy and led to the turnover and extinction of many endemic species, facilitating the diversification of the colonizing lineages. As a primary example, South America harbors the most diverse extant canid community, with 10 currently recognized species (2), including disparate forms such as the squat bush dog and the long-legged maned wolf (Fig. 1). Due to their rapid and relatively recent diversification, three fundamental aspects of their evolutionary history remain uncertain.

First, the pattern of invasion and dispersion of canids into South America is poorly defined. Although it is widely agreed that the first canids migrated into South America using the Panama land bridge, when this migration event occurred is uncertain. Molecular, fossil, and geological data suggest that several terrestrial dispersal events may have occurred prior to the generally cited estimate of approximately 3 Mya for the complete formation of the land bridge (3–5). There is also uncertainty about the number of ancestral lineages that entered South America and the antiquity of their diverse adaptations (6–13). The Andes were close to their present-day elevation 6 Mya in the Late Miocene (14). Therefore, when canids arrived in South America, the Andes formed a distinct geographic barrier that extended along the length of the continent (9, 13, 15–19). Although canids are found on both sides of the Andes, the ancestral canid lineage could have entered the east or west side of the Andes or both sides simultaneously and subsequently diversified. Knowing the number of invasions and dispersion of canid lineages in South America is critical to understanding the timing, interrelationships, and environmental correlates underlying this burst of speciation.

Second, the extent and timing of speciation and lineage-specific demographic history, including the potential impact of substantial climatic differences on each side of the Andes, are not well understood. Along the eastern lowlands, the glacial periods of the Pleistocene led to an expansion of savanna vegetation into areas that now support forest (20–23). To the west, glacial periods caused the expansion of glaciers in the south and shifts of vegetation zones along the Andean slopes (24–26). These changes likely reduced the availability of favorable habitats for some canid species but extended the distribution of others (20, 27, 28). Currently, the Andes restrict the geographic range of western species, leaving only a relatively narrow belt between the mountains and the Pacific Ocean (29).

Finally, the genetic complexity of the unique adaptations observed in the bush dog (*Speothos venaticus*) and the maned wolf (*Chrysocyon brachyurus*), sister species with extraordinary morphological differences (30–36), has not been explored. The bush dog is the only obligate meat-eating (hypercarnivorous) canid in the Americas that survived the Late Pleistocene extinction (11, 36). It has a suite of dental and skeletal specializations that are linked to its ability to capture and process prey (37–39). These features include short robust legs with webbed feet to dig burrows for hunting and shelter, long bodies with short tails, a unique extension of the meat cutting blade in the upper fourth premolar (P4) and lower first molar (M1) teeth (a trenchant heel), and loss of molars which in other canids function to crush hard plant foods (36, 38–40). The maned wolf is the only large-bodied canid in South America to survive the Late Pleistocene megafaunal extinctions, which may reflect its ability to exploit a wide range of foods in the savanna-like Cerrado environment, including a large proportion of fruits in its diet (30, 31, 41–43). Furthermore, the maned wolf has the longest limbs among canids (Fig. 1) but is not a swift runner (31, 35), as its speed is limited by its unique racking gait characterized by lifting both feet on each side of the body simultaneously, facilitating movement through tall grassland (30, 44). Although certain aspects of the biology of bush dogs and maned wolves have been extensively studied (30–36), the genomic underpinnings of their extraordinary adaptations remain unknown.

We investigated the evolutionary history of South American (SA) canids using newly generated whole-genome sequence data to assess phylogenetic relationships, evidence of interspecies gene flow, demographic history, and patterns of genomic diversity. To investigate adaptive evolution and genome variation in the maned wolf and bush dog, we generated de novo genome assemblies for both species and additionally sequenced four maned wolves and three bush dogs to characterize patterns of genetic diversity in the wild.

## Results

We generated whole-genome sequencing reads for 18 SA canids, ranging from 27.4 Gb for the pampas fox to 98.8 Gb for the bush dog, which were combined with 13 previously published genomes representing 22 species (*SI Appendix*, Table S1). Approximately 98% of these reads successfully mapped to the domestic dog reference genome. The mean coverage for the SA canid genomes ranged from 11.78× in the pampas fox to 77.7× in the maned wolf (*SI Appendix*, Table S1). Additionally, we constructed de novo assemblies of one bush dog and one maned wolf to avoid any reference bias in our positive selection analyses. The GC content of both genomes was 41%. The bush dog assembly had a contig N50/L50 of 40,867/16,741 bp and scaffold N50/L50 of 571,622/1,209 bp, and the maned wolf assembly had a contig and scaffold N50/L50 of 62,925/10,606 bp and 739,658/972 bp, respectively. We obtained 3,799 complete (91.5%) and 200 fragmented (4.9%) benchmarking universal single-copy orthologs (BUSCOs) for both assemblies, which also contained 27% of repetitive sequence (*SI Appendix*).

### Biogeographic History.

We found that SA canids are monophyletic, with one subclade representing the genus *Lycalopex*, another comprising the bush dog and maned wolf, and a third subclade containing the crab-eating fox (*Cerdocyon thous*) and the enigmatic short-eared dog (*Atelocynus microtis*) (Fig. 1). These phylogenetic relationships were reconstructed with ASTRAL-III (45) using all 10 extant SA species and 12 other species from the genera *Canis*, *Lupullela*, *Lycaon*, and *Cuon* (Fig. 1 and *SI Appendix*, Fig. S1).

Given the potential influence of the Andes on the diversification of SA canids, we reconstructed the ancestral distribution of the extant species using the R package BioGeoBEARS (46) (*Materials and Methods*). We tested three different models to estimate the distribution of hypothetical ancestors (internal nodes) and shifts across the west, center, or east of the Andes along the phylogeny: dispersal–extinction–cladogenesis (DEC); dispersal vicariance analysis (DIVALIKE); and the Bayesian analysis of biogeography (BAYAREALIKE). Additionally, we tested the same three models plus a founder effect parameter on each model named “J”: DEC+J, DIVALIKE +J, and BAYAREALIKE+J (46). Among these six tested models, DIVALIKE+J best fit our data (*SI Appendix*, Table S2). According to this model (*SI Appendix*, Table S2 and Fig. S2), we found a 90% probability that the lineages ancestral to the bush dog, maned wolf, crab-eating fox, and short-eared dog originated east of the Andes (nodes 14, 15, 23, and 24 in Fig. 1). Further, the model estimates a 60% probability that the hoary fox also originated east of the Andes (node 22 in Fig. 1). The model suggests that, after the divergence of the hoary fox, an ancestral SA fox species expanded from east to the west of the Andes with 65% probability (node 21 in Fig. 1). This result is consistent with a scenario where canids circumvented the Andes along their southern edge. Following this range expansion, two ancestral SA fox lineages arose on opposite sides of the Andes. One originated west of the Andes with 99% probability and subsequently diverged into Darwin’s fox, the Sechuran fox, and the culpeo fox (*Lycalopex culpaeus*) (nodes 19 and 20). The other lineage gave rise to the pampas fox (*Lycalopex gymnocercus*) and SA gray fox (*Lycalopex griseus*) on the eastern side of the Andes with a probability of 62% (node 17).

### Species Divergence and Interspecific Gene Flow.

**Fig. 2.**
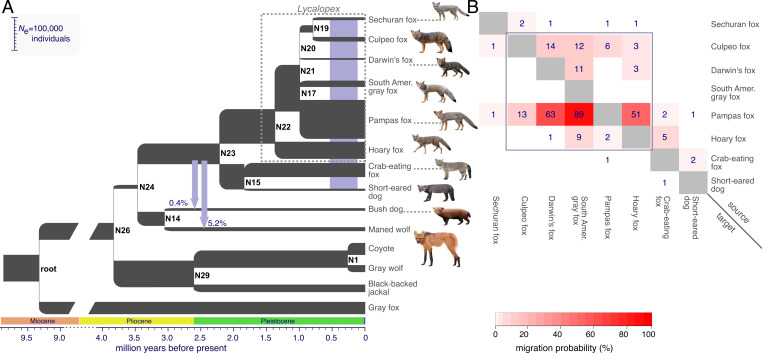
(*A*) Graphical summary of the demographic model estimated using G-PhoCS. The branch widths and lengths are scaled proportionally to inferred effective population sizes and species divergence times, respectively. Parameter scaling assumes an average per-generation mutation rate of μ = 4.0 × 10^−9^ and an average generation time of 4 y. The purple vertical bar along the terminal branches indicates evidence of gene flow found in 25 directed migration bands among the eight SA canids in that clade (*B*). Two additional ancestral migration bands with significant rates are shown as vertical arrows. Node labels, as in the assumed species tree (Fig. 1), are specified to the *Right* of each divergence event. (*B*) Probabilities of migration between pairs of SA foxes. Migration probabilities within the genus *Lycalopex* are shown inside the purple square (*SI Appendix*, Table S3).

We inferred a complete demographic model for SA canid species, including species divergence times, ancestral population sizes, and interspecific gene flow. We assumed the species tree inferred with ASTRAL-III and applied G-PhoCS (47) to the genome sequences of 14 canid species (*Materials and Methods*). The inferred demographic history suggests that the species ancestral to all SA canids lived between 3.9 Mya and 3.5 Mya and had a relatively small effective population size of ∼11,600 individuals (branch between nodes 24 and 26 in Fig. 2*A* and *SI Appendix*, Table S3). The bush dog and maned wolf diverged shortly after this time, approximately 3.1 Mya (node 14 in Fig. 2*A* and *SI Appendix*, Table S3), with little gene flow as indicated by low migration probabilities (≤5.2%) into these two lineages. The six *Lycalopex* species diverged within a fairly short time period between 1.43 Mya and 0.81 Mya. The Sechuran fox appears to be the most genetically isolated among the six species, with substantially higher levels of gene flow inferred for the other five species (Fig. 2*B*). The pampas fox appears to be an especially prominent source of gene flow, as suggested by very high migration probabilities (>50%) inferred from this species to the eastern hoary fox, the western Darwin’s fox, and the SA gray fox. Thus, our results suggest that the speciation of the deeper lineages is more complete, whereas the recent diversification of *Lycalopex* involved a higher degree of interspecific gene flow during their radiation (Fig. 2 *A* and *B*). Supporting this observation, we found that phylogenetic discordance was the greatest in *Lycalopex*, the group with the most extensive interspecific gene flow (*SI Appendix*, Fig. S3).

### Genetic Diversity and Runs of Homozygosity.

**Fig. 3.**
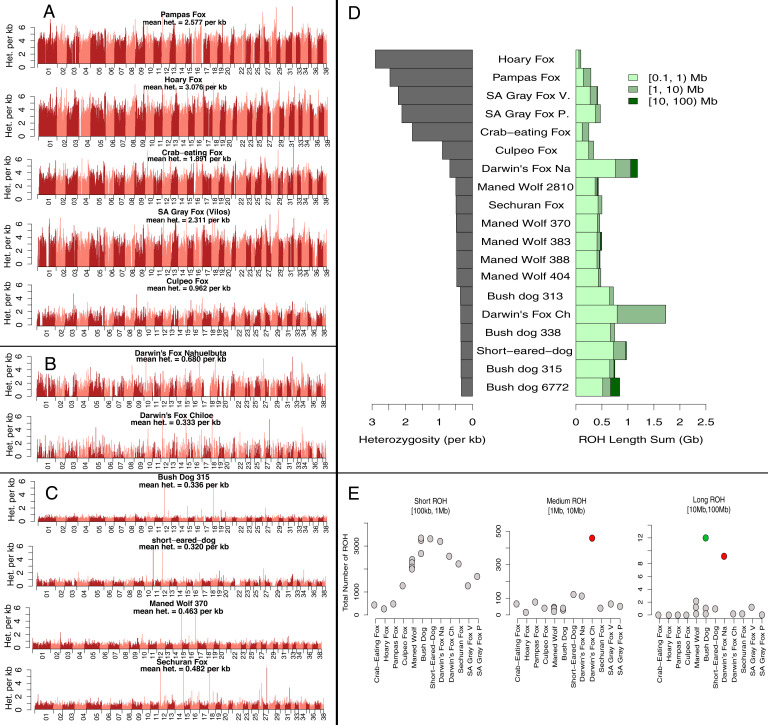
Heterozygosity computed using 100-kb windows with a 10-kb step size across the genomes of SA canids. Three different patterns were found: (*A*) Species with high heterozygosity; (*B*) species with high heterozygosity alternating with long stretches of depleted genetic diversity; and (*C*) species with low heterozygosity. (*D*) Per-site autosomal heterozygosity for 19 SA canid genomes (*Left*) and summed lengths of ROH of three specific length categories: short (0.1 Mb to 1 Mb), medium (1 Mb to 10 Mb), and long (>10 Mb) (*Right*). (*E*) The total number of ROH in the three length categories. Darwin’s fox from Chiloé Island and Nahuelbuta are shown as red dots. The bush dog sampled from the Little Rock Zoo is shown as a green dot.

We observed three patterns of genome-wide heterozygosity across nonoverlapping 100-kb windows among SA canids (Fig. 3 *A–C*). First, the five open/mixed habitat species, crab-eating fox, hoary fox, pampas fox, SA gray fox, and, to a lesser degree, culpeo fox, had high heterozygosity across most autosomes (Fig. 3*A*). Second, Darwin’s fox had regions of high heterozygosity alternating with homozygous stretches that spanned nearly entire chromosomes (Fig. 3*B*). For instance, chromosome 17 was practically depleted of heterozygosity in Darwin’s fox from Nahuelbuta National Park, Chile. Lastly, four species (short-eared dog, bush dog, maned wolf, and Sechuran fox) had genomes with uniformly low heterozygosity across most autosomes (Fig. 3*C*).

To further assess the regions of the genome that exhibited depleted genetic variation (Fig. 3 *B* and *C*), we quantified runs of homozygosity (ROH) (Fig. 3 *D* and *E* and *SI Appendix*, Table S4). The Darwin’s fox from Nahuelbuta National Park, Chile, had more long ROH (>10 Mb) than any of the other wild canid (Fig. 3 *D* and *E*), consistent with a history of a recent population bottleneck and inbreeding (48). These long ROH totaled 131 Mb (5% of the genome), as opposed to an average of 18 Mb (<1% of the total genome) in the other nine species, excluding the likely inbred zoo-sampled bush dog (Fig. 3*D*). Similarly, Darwin’s fox from Chiloé Island, located off the coast of southern Chile (Fig. 3 *D* and *E* and *SI Appendix*, Table S4), had a high amount of ROH. The total length of medium ROH in Darwin’s fox from Chiloé Island was about four times greater than any other canid, accounting for 936 Mb or 37% of its genome (Fig. 3 *D* and *E* and *SI Appendix*, Table S4). These results are consistent with the low census estimates for Darwin’s fox (49) and with its exceptionally low effective population size inferred with G-PhoCS (5,100 to 5,500 Bayesian 95% CI; Fig. 2*A* and *SI Appendix*, Table S3).

The bush dog, short-eared dog, maned wolf, and Sechuran fox had genomes dominated by short ROH, which averaged 512 Mb in these species (*SI Appendix*, Table S4), and represented ∼20% of the genome. In addition, these species were characterized by medium (1 to 10 Mb) and long (>10 Mb) ROH that represented 3.4% and 0.96% of the genome, respectively. Accordingly, these four species had relatively low heterozygosity (<1 heterozygote site/kb) and small effective population sizes ranging from 6,200 to 12,400 (Figs. 2*A* and 3 *D* and *E* and *SI Appendix*, Table S3). Species with high heterozygosity (>1 heterozygote site/kb), including the crab-eating, hoary, pampas, and culpeo foxes (Fig. 3 *D* and *E* and *SI Appendix*, Table S4) had fewer ROH, which averaged <1 Mb in length, covered 6% of the genome, and had no ROH >10 Mb (Fig. 3 *D* and *E*). These results are consistent with the large effective population sizes inferred for these species, ranging from 13,300 to 19,900 in the culpeo fox and SA gray fox and from 41,900 to 122,600 in the remaining three species (Fig. 2*A* and *SI Appendix*, Table S3).

### Demographic Analysis.

**Fig. 4.**
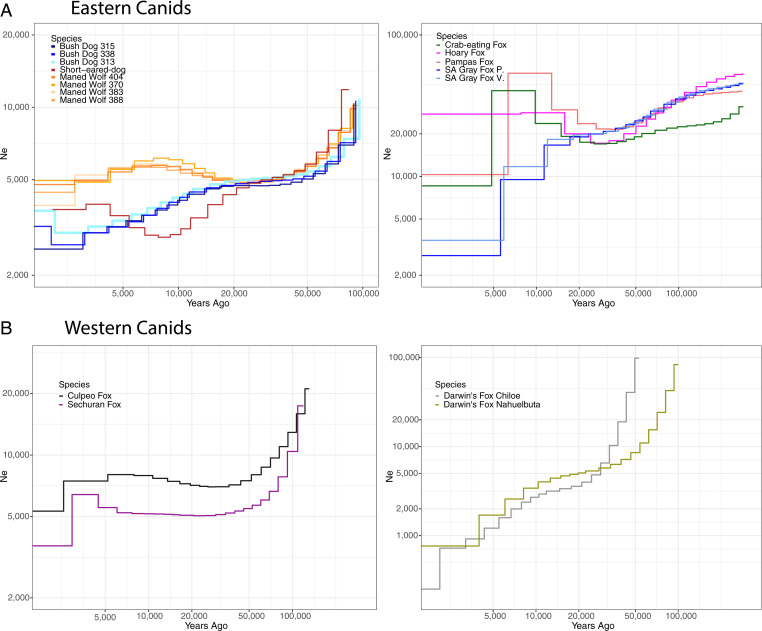
Demographic history of South American canids inferred using MSMC (114). The *y* axis corresponds to inverse instantaneous coalescent rates (IICRs) scaled by 2µ, which is a proxy for the effective population size (*N*_e_) through time. IICRs were scaled to 4-y generation time and a mutation rate of 1.009 to 08.

We observe contrasting demographic trajectories during the past 100,000 y for SA canids (Fig. 4). We first examined eastern species that showed low genetic diversity in our previous analyses. Among these species, the maned wolf showed a stable but low *N*_e_ trajectory of ∼5,000 (Fig. 4 *A*, *Left*), whereas the bush dog and short-eared dog exhibited a gradual decline in *N*_e_ over the past 20,000 y to the present-day levels of ∼3,000. Notably, in the bush dog, a separate analysis that focused on functionally relevant mutations showed that it had an average of 227 damaging homozygote-derived genotypes, roughly 30% more than other SA canids (*SI Appendix*, Fig. S4 and Table S5 and see *SI Appendix*). Additionally, the short-eared dog appears to have experienced a more dramatic decline than other SA canids, followed by a recent recovery. However, this trajectory could also reflect historic population structure (50, 51). The other eastern species showed relatively large and stable effective sizes of ∼20,000, with the SA gray fox showing a decline in *N*_e_ during the past 20,000 y (Fig. 4 *A*, *Right*). For western species, the Sechuran and culpeo foxes had a sharp decline ∼50,000 y ago followed by a long-term stable effective population size of 5,000 and 8,000, respectively (Fig. 4 *B*, *Left*). In contrast, the effective population size of Darwin’s fox declined steadily over the past 50,000 y, reaching present-day levels of <1,000 (Fig. 4 *B*, *Right*). Overall, the *N*_e_ trajectories produced by MSMC (multiple sequentially Markovian coalescent) refine the coarse-grained estimates based on G-PhoCS and genomic diversity (Figs. 2 and 3), suggesting that low diversity in Darwin’s fox and the bush dog is due to gradual ongoing species decline, whereas the Sechuran fox and short-eared dog have experienced more ancient declines that have since stabilized.

### Genetic Basis of Bush Dog and Maned Wolf Adaptations.

We found signals of positive selection in both coding and regulatory regions in bush dogs and maned wolves, which are the most disparate species concerning ecology and morphology despite having shared a common ancestor of only 3.1 Mya (Fig. 2*A* and *SI Appendix*, Table S3). We examined positive selection in a three-layered approach, avoiding reference bias by mapping every candidate gene mutation to the de novo assembly of the bush dog and maned wolf and checking for inconsistencies.

First, to identify amino acid changes that could have undergone positive selection in the bush dog or maned wolf, we analyzed a panel of 17,181 genes annotated in the domestic dog genome (*Materials and Methods*). After stringent filtering, visual inspection, and confirmation of identified mutations in the de novo assemblies of the two species, we identified two genes in the bush dog (*MAPK8IP3* and *TRAF1*) and one in the maned wolf (*BICRA*) as significant after correction for multiple hypothesis testing (q value <5%). Although *MAPK8IP3* and *TRAF1* have not previously been associated with adaptive evolution, *BICRA* has been linked with dysmorphic craniofacial features (52) and could be associated with the relatively long snout of the maned wolf (*SI Appendix*, Tables S6 and S7). Despite not being significant after multiple hypothesis correction, *Beta-1,4-Galactosyltransferase 7* (*B4GALT7*) was among the genes with the highest evidence of positive selection (*P* < 0.01) in the short-legged bush dog. Interestingly, this gene is involved in the synthesis of sulfate proteoglycans (heparan and chondroitin), which contribute to limb elongation through transportation of Indian hedgehog (Ihh) during chondrocyte proliferation (53, 54). Recessive mutations in *B4GALT7* are directly linked to dwarfism in humans and Friesian horses (55, 56), and have been associated with the short stature phenotype in spondylodysplastic Ehlers-Danlos syndrome (57).

**Fig. 5.**
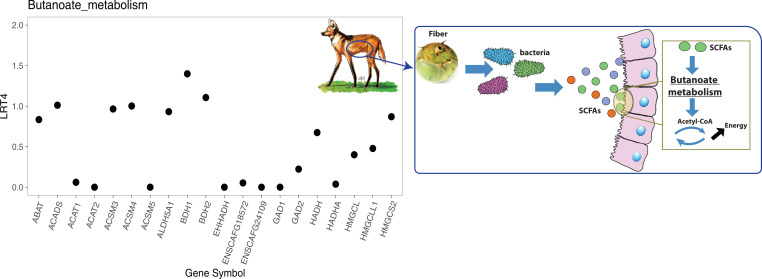
Adaptation to digest fruit fiber in the maned wolf. (*Left*) Genes found to be enriched in the butanoate metabolism category, as inferred by polysel (58). The *y* axis indicates the fourth root of the likelihood ratio test statistic score (LRT4), which resulted in an increasing score (q = 0.01) for the butanoate metabolism category. Gene labels are shown on the *x* axis. (*Right*) Schematic of fruit fiber digestion and the role of the candidate metabolic process.

Morphological and dietary differences between the maned wolf and bush dog were likely driven by many genes. Since these genes could collectively regulate multiple biological pathways, each might have a moderate or weak signal of positive selection. Therefore, we used polysel (58) to explore the possibility of polygenic signals of selection on the branches leading to the two species, assessing patterns in biological pathways related to frugivory in the maned wolf and hypercarnivory in the bush dog and to limb elongation in both species (*SI Appendix*, Tables S8 and S9). Polysel identified as significant (q < 0.05) butanoate metabolism in the maned wolf, which is related to energy intake from short-chain fatty acids (SCFAs) derived from fruit fiber (Fig. 5 and *SI Appendix*, Table S8). To test whether butanoate metabolism is uniquely associated with amino acid changes in the maned wolf, we further evaluated gene ontology (GO) categories associated with frugivory in other canid species, including gray wolf, dhole, and African wild dog (*SI Appendix*, Table S10). As expected, butanoate metabolism was not significant in other canids, which further supports its association with the fruit-rich diet of the maned wolf.

To test the effect of mutations at the protein level, we calculated the sorting intolerant from tolerant (SIFT) scores with the variant effect predictor (VEP) tool (59) and identified in-frame indels, frameshift variations, and stop codon gains unique to either the bush dog or maned wolf that could have a significant effect on their associated proteins. We focused on mutations with a putative high impact on the proteins, as these are most likely to have been subjected to selection. We identified 656 genes in the bush dog with unique, high-impact mutations, including a unique two-nucleotide insertion in the 3′ UTR region of the *MSX1* gene, which affects gene expression in both tooth and digit development (60, 61). We also found mutations in *SULF2*, a gene responsible for sulfation of the receptor heparan sulfate proteoglycan (62). This result is consistent with our finding on *B4GALT7* described above, of genetic variants in regulatory regions associated with chondrocyte proliferation through the synthesis of chondroitin sulfate proteoglycans (53, 54). We identified 450 high-impact genetic variants unique to the maned wolf, including a loss of a stop codon in the alcohol dehydrogenase 4 gene (*ADH4*). This gene facilitates energy transfer in the form of NADH when converting alcohol to ketone.

**Fig. 6.**
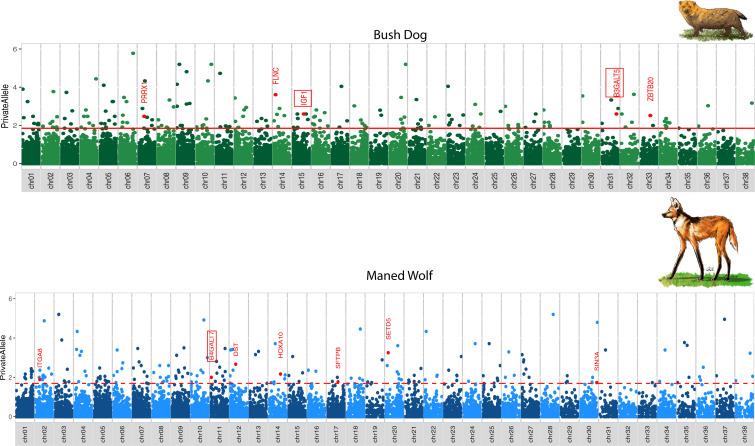
Proportion of private alleles for the bush dog (*Top*) and maned wolf (*Bottom*) from 1-kb windows flanking 39,704 transcripts belonging to 32,704 genes. Significant genes (*P* value <0.01) are shown above the horizontal red line. Genes involved in limb development are shown in red. Genes relevant to limb elongation are highlighted by a red rectangle.

Genetic changes associated with limb elongation in mammals have been found principally within regulatory regions (63, 64). Therefore, we examined promoter and enhancer regions otherwise conserved in the Canidae that had lineage-specific changes in the bush dog or maned wolf. We analyzed 1-kb windows flanking the start and end sites from 39,704 transcripts belonging to 32,704 genes from the domestic dog genome (CanFam3.1). Within each window, we calculated the number of private alleles for the bush dog and maned wolf as well as the average number of variable sites across the remaining species. We found 110 genes in proximity to these windows with *P* values below 0.01 (Fig. 6). Of these, 12 genes have known direct or indirect associations with limb development (Fig. 6 and *SI Appendix*, Tables S11 and S12). Notably, *IGF1*, *B3GALT5*, and *B4GALT7* are related to chondrocyte proliferation through the synthesis of sulfate proteoglycans and chondrocyte enlargement (53, 54, 65) and are primary genes associated with limb diversification in mammals (66). Moreover, the coding sequence of *B4GALT7* appears to be under selection in the bush dog (see above), and its regulatory region has a signal in the maned wolf as well (*SI Appendix*, Tables S6 and S12). Finally, we found that the bush dog and maned wolf had an overall higher proportion of private alleles in regulatory regions of genes involved in endochondral bone elongation via chondrogenesis compared to the eight other canid species tested (*SI Appendix* and *SI Appendix*, Table S13).

## Discussion

### The Geography of a Rapid Radiation.

We reconstructed population sizes of ancestral lineages to better understand canid colonization of South America. Our estimates showed that the ancestor of all SA canids existed 3.9 to 3.5 Mya and had a relatively small population size of 11,600 (95% Bayesian credible interval = 10,900 to 12,400) (Fig. 2*A*). This result is consistent with a dispersal event through the developing Panamanian corridor of Central America between the late Pliocene and early Pleistocene (4, 67, 68). This corridor was narrow and primarily covered with savanna, presumably limiting population sizes and acting as a selective filter (67, 68). On reaching South America, canids encountered a vast and diverse landscape with few obligate carnivores, which were functionally distinct, such as marsupials and terror birds (Phorusrhacidae) (69). Thereafter, our G-PhoCS results are consistent with a fivefold increase in the population size of the ancestor of SA foxes and the common ancestor of the bush dog and maned wolf about 3.5 Mya (Fig. 2*A* split after node N24; *SI Appendix*, Table S3), followed by an increase in the diversification of SA foxes at 2.2 Mya (Fig. 2*A* at node N23; *SI Appendix*, Table S3). SA canids are an example of an extremely rapid radiation not approached by other extant clades of large-bodied carnivorans.

Our findings of a single dispersal of canids into South America between 3.9 and 3.5 Mya is consistent with previous molecular studies (9). However, this model has been challenged by the presence of North American fossils assigned to the maned wolf (*Chrysocyon*) and crab-eating fox (*Cerdocyon*) lineages from the early Pliocene, ∼5 Mya (11, 70, 71), suggesting that these groups predate the closure of the Panamanian land bridge (3, 4). Nonetheless, these fossil remains are fragmentary, and correct phylogenetic assignments have proven difficult, precluding their use as conclusive evidence against a single-dispersal model (*SI Appendix*).

Following immigration into South America via the Panamanian land bridge, our biogeographic model showed that canids first diversified east of the Andes (Fig. 1), which suggests that they used a natural corridor known as the “savanna route” to disperse from Central America to eastern South America (13, 67, 72). This route was followed by many mammals during this time period, including felids, procyonids, large horses, and large proboscideans, and suggests that the Andes may have been a barrier to the western dispersal of most mammals during the early stages of the Great American Biotic Interchange. Subsequently, canids diversified in a north–south pattern along the eastern regions leading to the development of the bush dog, maned wolf, crab-eating fox, short-eared dog, and hoary fox (Fig. 1). Our results indicate that an ancestral *Lycalopex* lineage dispersed to west of the Andes 1.0 Mya (Figs. 1 and 2*A*, node N21), probably during the dry periods of the Pleistocene when savannas expanded in South America (20–22). Given that the oldest fossil record of *Lycalopex* was found in austral regions (11, 73), it is likely that this ancestral lineage circumvented the Andes in southern Patagonia to colonize the western side of the continent. Multiple species then diversified from this ancestral lineage, including the Pampas fox and SA gray fox in the east and Darwin’s fox, culpeo fox, and Sechuran fox in the west (Fig. 1). This burst of speciation is consistent with the fossil record suggesting that the center of the *Lycalopex* radiation was Argentina (11) and establishes the Andes as a critical constraint on the early diversification of SA canids.

### Interspecific Gene Flow and Changing Habitat Dynamics.

Our biogeographic scenario predicts that species resulting from early divergences in eastern South America should have less interspecific gene flow than recently diversified species on the western side of the Andes. The migration probabilities estimated with G-PhoCS partially support this prediction. Specifically, migration probabilities for most species west of the Andes suggest >10% of ancestral lineages originated from another species by gene flow. In contrast, species on the eastern side of the Andes, including the maned wolf, bush dog, short-eared dog, and crab-eating fox, that diverged more than 1.9 Mya, had migration probabilities smaller than 5% (Fig. 2*A*). The only eastern species that experienced considerable levels of gene flow were of the genus *Lycalopex*, and within this group, most of the detected gene flow derived from the Pampas fox to other species (Fig. 2*B*). The extremely high probability of migration (90%) from the Pampas to the SA gray fox suggests that these two species have been almost freely mixing at various times in their evolutionary history. Indeed, these species are morphologically similar and have been suggested to be conspecific (74, 75). A very high migration probability (51%) is also inferred from Pampas to hoary fox (Fig. 2*B*) and likely reflects an ancient shared history of these two species rather than recent admixture, since our samples derive from locations outside any known hybrid zone (*SI Appendix*, Table S1) (19, 76). The extensive ancestral gene flow from Pampas fox to SA gray fox and hoary fox likely contributed to their elevated genetic diversity (Fig. 3*D*). Moreover, interspecific gene flow during the radiation of *Lycalopex* foxes possibly augmented their adaptive potential through the introduction of new alleles, facilitating their rapid ecological diversification (74, 75, 77–81), as in other species (82–84).

### Population Size of SA Canids.

Different paleoclimatic conditions on each side of the Andes may have shaped the demographic history of SA canids. The expansion of ice sheets was likely the most important influence on population size on the western side, whereas the expansion of savannas was critical in the east (20–23). Supporting this prediction, our MSMC models showed a decrease in population size of the short-eared dog and bush dog ∼20 kya, both eastern species that occur in forest habitats, although the latter is also found in savannas and wetlands (37–39) (Fig. 4*A*). Concurrently, we observed an increase in the population size of fox species that prefer open habitats (hoary fox, SA gray fox, Pampas fox) or are habitat generalists (crab-eating fox). The population size increase of open-habitat species coupled with a decline of forest-dwelling canids could be related to environmental changes that occurred during the Last Glacial Maximum (LGM), 19 to 26.5 kya (85). During this time, there was a 25% reduction in rainfall, which led to a 34 to 67% expansion of grasslands and a 14 to 25% reduction of forests (21, 22). These findings suggest that expansion of grasslands may have permitted open-habitat canids to extend their range and increase their population size, likely outcompeting forest-dwelling species as their habitat contracted.

For Darwin’s, Sechuran, and culpeo foxes found west of the Andes, our MSMC models suggest relatively small population sizes (Fig. 4*B*). The Andes restricted the geographic range of western species, leaving a narrow belt between them and the Pacific Ocean, reducing potential population sizes as the lowlands west of the Andes are only 3% of the width of the eastern lowlands (29). For example, the Sechuran fox has a minute distribution in northern Peru (86). In contrast, the culpeo fox has the largest geographic range and population size (87, 88) and the highest heterozygosity among the western species. These results further establish the Andes as an important driver of genomic variability and adaptive divergence.

### Conservation of South American Canids.

Our genomic results on Darwin’s fox suggest that recent population declines and inbreeding (89–91) may have contributed to the depletion of genetic diversity and genomes with long ROH, especially in the mainland individual (Fig. 3 *B*, *D*, and *E*). Population size decreases reflect a reduction of temperate forest habitat as well as the introduction of domestic dogs, which may have led to their near elimination on the mainland, with only ∼78 individuals remaining (92). Captive breeding and habitat restoration are urgently needed to allow mainland populations to expand to parts of their previous geographic range and to retain genetic viability (*SI Appendix*).

Among other canids, the short-eared dog, bush dog, and Sechuran fox had the lowest genome-wide heterozygosity (*SI Appendix*, Fig. S5), with values similar to that observed in endangered canids such as the Ethiopian wolf, African wild dog, and dhole (*SI Appendix*, Fig. S5). The bush dog had the highest proportion of “damaging” variants (*SI Appendix*, Fig. S4), which could be related to their long-standing small population size (*SI Appendix*). Due to the bush dog’s apparent genetic load, the status of the remnant population is a pressing conservation concern. Habitat restoration may be critical to expanding their population size and increasing gene flow.

### Genetic Basis of Bush Dog and Maned Wolf Adaptations.

Although the bush dog and maned wolf have been the focus of ecological and morphological studies, the genomic mechanisms underlying their unique adaptations have remained a mystery (11, 12, 30, 31, 33, 34, 36, 93, 94). The maned wolf is the only large canid having a diet consisting largely of fruits, including a tomato-like fruit commonly known as the “wolf apple” (*Solanum lycocarpum*) in Brazil (30, 31, 41, 42) and wild guava, *Alibertia edulis* (Rubiaceae) in Bolivia (43). These fruits contain high amounts of polysaccharides that are indigestible for most carnivores (95, 96). In mammals, including canids, polysaccharides are often broken down by bacterial fermentation in the cecum (97, 98). Given that maned wolves lack a long intestine to make extensive fermentation possible (94), they likely have physiological adaptations that allow them to process bacterial products more efficiently. Our finding of polygenic signals of selection related to energy intake from SCFAs provides a mechanism by which maned wolves digest complex sugars. Specifically, SCFAs are processed from polysaccharides derived from fruit fiber in the cecum during bacterial fermentation (97) and unique mutations in the maned wolf could enhance the transformation of SCFAs into an energy precursor, acetyl-CoA, through the butanoate metabolism process (Fig. 5). Once acetyl-CoA is produced, it releases energy in the form of adenosine triphosphate in the citric acid cycle (97, 98).

The bush dog and maned wolf have the most divergent body size and limb proportions of any closely related canids, which may represent divergent adaptations to facilitate burrowing for hunting and denning by bush dogs and for navigation through tall grasslands by maned wolves (30, 37, 38). Differences in limb length at birth are not explained by variation in gestation time (33, 34). Therefore, a molecular pathway that decreases the rate of fetal limb growth while not affecting the growth of the axial skeleton may exist, such as in various chondrodysplasias (33, 34, 99). We find unique mutations in regulatory regions and amino acid changes that may affect chondrocyte proliferation and enlargement when chondrocytes multiply in the proliferation region (*SI Appendix*, Fig. S6 *D* and *J*) and subsequently, when these cells grow to become hypertrophic chondrocytes and triple in size (65, 66) (*SI Appendix*, Fig. S6 *E* and *K*).

Genomic windows enriched with lineage-specific alleles proximate to *B4GALT7* in the maned wolf and *B3GALT5* in the bush dog are associated with limb elongation through the synthesis of the sulfate proteoglycans heparan and chondroitin (53). Interestingly, *B4GALT7* was also enriched with amino acid mutations unique to the bush dog, which suggests that this gene appears to have evolved through functional changes in coding and regulatory regions. Additionally, the variant in *SULF2* that we found in the bush dog may also have a significant impact on the sulfation of heparan sulfate proteoglycans (62). Sulfate chondroitin proteoglycans (CSPG) and heparan sulfate proteoglycans (HSPG) transport Indian Hedgehog (Ihh) from the hypertrophic region to the top region of the proliferation zone (*SI Appendix*, Fig. S6 *B* and *H*) (54). In this region, Ihh activates target genes necessary for chondrocyte multiplication (*SI Appendix*, Fig. S6 *C* and *I*). The rate at which new chondrocytes are generated is determined by the proportion of CSPG and HSPG (54). Our findings imply that a relatively high rate of sulfation of chondroitin proteoglycans may promote longer limbs in the maned wolf (*SI Appendix*, Fig. S6 *A–F*), whereas lower sulfation may result in short limbs in the bush dog (*SI Appendix*, Fig. S6 *G–L*) (54). The other gene relevant to limb proportion is *IGF1*, which is significantly enriched with private alleles in the bush dog and determines the increase of hypertrophic chondrocytes through fluid intake and mass growth (*SI Appendix*, Fig. S6 *E* and *K*) (65). In the jerboa, *IGF1* is associated with a 40-fold increase in metatarsal cell volume (65). Similarly, this gene is related to enlargement of forelimbs in newborn opossums (100). In domestic dogs, one haplotype of *IGF1* explains 15% of size variance in different breeds (101, 102). Given the characteristic short limbs of bush dogs, our results suggest that unique mutations may decrease the expression of *IGF1* and consequently limit chondrocyte elongation (*SI Appendix*, Fig. S6*J*). A depletion of IGF1 reduces the duration of the cell enlargement process, thus restricting limb elongation (*SI Appendix*, Fig. S6 *K* and *L*) (65). Supporting this view, mice deficient in IGF1 failed to double the volume of their chondrocytes, resulting in short limbs (103).

The other unusual characteristics of the bush dog include interdigital webbing that enhances digging abilities and extraction of prey such as armadillos from burrows (37–39). Bush dogs also uniquely demonstrate molar reduction and a trenchant heel on the lower carnassial teeth (*P*_4_/M_1_) that improves meat cutting (36). No other SA canid has these dental features, which in other carnivores are associated with an emphasis on meat rather than plant material in the diet (36). Our VEP analysis suggests a candidate gene, *MSX1*, may be related to molar reduction and interdigital webbing. We found a 2-bp insertion at the 3′ UTR region of *MSX1* that, among canids, is unique to the bush dog and likely affects gene translation. Previous studies have shown that mutations in this region can affect the functionality of *MSX1* (104, 105), which in turn influences tooth development (104). In particular, a decrease in MSX1 reduces bone morphogenetic protein 4 (BMP4), which lowers the proliferation of mesenchymal cells during tooth formation (106). In the case of digit development, a decrease of *MSX1* expression increases the effect of its antagonist, *FGF8*, which prompts the survival of mesodermal cells in the interdigital web (60, 61). Therefore, a decrease in the expression of *MSX1* potentially has a dual phenotypic effect in the bush dog, specifically, a loss of molars in tooth development and interdigital web formation. If confirmed by experimental data, this dual effect would offer a striking example of morphological innovation involving pleiotropic changes in a single gene. Overall, our results reveal evolutionary insights into the adaptive radiation of the most diverse community of endemic canids, which have puzzled evolutionary biologists and naturalists for centuries.

## Materials and Methods

We extracted and sequenced genomic DNA from 18 SA canids and downloaded the genomes of 13 other individuals from the National Center for Biotechnology Information (NCBI)’s Sequence Read Archive (*SI Appendix*, Table S1). We filtered and mapped raw reads to the domestic dog CanFam3.1 (107) and conducted genotype calling using a modified pipeline from the Genome Analysis Toolkit (108) (*SI Appendix*). We de novo assembled the bush dog and maned wolf sequencing reads using MaSuRCA version 3.3.3 (109), extendeding K-mers with a modified version of the Celera Assembler (CABOG) (110). We calculated basic statistics of the de novo assemblies with assembly_stats version 0.1.4 (111) (*SI Appendix*, Tables S14 and S15). We reconstructed a species tree with ASTRAL-III (45) and calculated average genomic divergence times with MCMCTree (112) using calibration priors (*SI Appendix*, Table S16) and generation times (*SI Appendix*, Tables S17 and S18). To investigate the geographic origin of extant SA canids, we used the R package BioGeoBEARS (46). To obtain a detailed demographic model for SA canids, we applied G-PhoCS v1.3.2 (https://github.com/gphocs-dev/G-PhoCS). We examined genome-wide site heterozygosity and quantified the extent of ROH in SA canids using PLINK (113). To investigate species demographic history, we used MSMC (114) with various mutation rates and generation times (*SI Appendix*, Fig. S7). We assessed the effects of nucleotide mutations in protein-coding genes from each of the 10 SA canid species using the VEP tool (59). To investigate positive selection, we used BioMart in Ensembl (Ensembl 104 release) to obtain the coordinates of 39,704 transcripts belonging to 32,704 genes from the CanFam3.1 domestic dog genome. After discarding genes with internal stop codons, lengths not multiple of three, and keeping the longest isoforms of each gene, we tested 17,181 orthologous genes (see Dataset S1 for a full list of genes tested) using the branch-site model in Codeml contained in PAML 4.8 (112). We used polysel (58) to detect biological pathways overrepresented by weak-to-moderate signals of selection in the bush dog and maned wolf. Finally, we assessed the enrichment of private alleles in the flanking regions of genes from the bush dog and maned wolf (*SI Appendix*, Fig. S8).

## Supplementary Material

Supplementary File

Supplementary File

## Data Availability

The de novo genome assemblies of the maned wolf and bush dog have been deposited in GenBank (https://www.ncbi.nlm.nih.gov) (115) under accessions JALPRZ000000000, version ChrBra_1.0 (https://www.ncbi.nlm.nih.gov/nuccore/JALPRZ000000000.1/), and JALPSA000000000 version SpeVena.1.0 (https://www.ncbi.nlm.nih.gov/nuccore/JALPSA000000000.1/) (116), respectively. The raw sequencing reads of the South American canid taxa generated for this study are available under BioProject PRJNA822671 (https://www.ncbi.nlm.nih.gov/search/all/?term=PRJNA822671) (117). Genomes from other canid taxa were obtained from previous publications and are available on NCBI (see Table S1 for accession numbers). The scripts used in this work are available on Github (https://github.com/dechavezv/2nd.paper.v2) (118). Previously published data were used for this work:(119), (120), (121), (122), (123), (124), (125), (126), (127), and (128).
