# Supplementary material for: Comparative genomics uncovers the evolutionary history, demography, and molecular adaptations of South American canids
Source: Proc Natl Acad Sci U S A. 2022 Aug 15;119(34):e2205986119. doi: 10.1073/pnas.2205986119 (PMC9407222; doi:10.1073/pnas.2205986119)
Supplement: Supplementary File [file pnas.2205986119.sapp.pdf]

**Supplementary Information**

## Comparative genomics uncovers the evolutionary history, demography and molecular adaptations of South American canids

Daniel E. Chavez, Ilan Gronau, Taylor Hains, Rebecca B Dikow, Paul B Frandsen, Henrique V. Figueiró, Fabrício S. Garcez, Ligia Tchaicka, Rogério C. de Paula, Flávio H. G. Rodrigues, Rodrigo S. P. Jorge, Edson S. Lima, Nucharin Songsasen, Warren E. Johnson, Eduardo Eizirik, Klaus-Peter Koepfli, and Robert K. Wayne

# Supplementary Material and Methods

### Genome sequencing

A whole blood sample was collected from a male maned wolf (studbook # 2810, NZP-CBI accession # 114862, DOB: 12/26/2005) housed in the Smithsonian's National Zoological Park-Conservation Biology Institute (NZP-CBI) in Front Royal, Virginia, during a routine veterinary check-up in 2014 and stored at -80ºC until genomic DNA extraction. Similarly, a whole blood sample was collected in 2015 from a female bush dog (studbook # 1410) housed in the Little Rock Zoo in Little Rock, Arkansas, during an annual veterinary check-up and shipped on dry ice to NZP-CBI and stored at -80ºC until genomic DNA extraction. We used the Qiagen DNEasy Blood and Tissue kit to extract genomic DNA from these two samples, which were then submitted to Psomagen, Inc. in Rockville, Maryland, and to the Vincent J. Coates Genomics Sequencing Laboratory at the University of California, Berkeley for genomic library preparation and sequencing. Two 350 bp-insert libraries and one 3 kb-insert size mate-pair library were

prepared for each sample using the Illumina TruSeq DNA PCR-free kit and the Nextera Mate Pair Library Preparation Kit, respectively. The libraries were quality checked using an Agilent Tapestation 4150 instrument and then sequenced on an Illumina HiSeq 2000 or HiSeqX instrument with 100 bp or 150 bp paired-end reads.

The raw reads from the bush dog and maned wolf were processed and then *de novo* assembled using MaSuRCA version 3.3.3 (1). MaSuRCA first corrects the Illumina reads using QuorUM (2). It then extends kmers with unambiguous extensions into longer "superreads," which are assembled into contigs and scaffolds with a modified version of the Celera Assembler (CABOG) (3). We used the program assembly_stats version 0.1.4 (4) to calculate the contig and scaffold statistics. The contig and scaffold N50/L50 for the bush dog were 40,867/16,741 bp (contig) and 571,622/1209 bp (scaffold) and for the maned wolf these were 62,925/10,606 bp (contig) and 739,658/972 bp (scaffold) (See Table S14 for details). Although the contiguity of both assemblies was relatively low, as expected from short-read sequencing reads, we retrieved sufficient orthologs with BUSCO (5), which allow us to control for reference bias in our analysis of positive selection. Out of 4,104 orthologs evaluated from the mammalia_odb9 gene set, we obtained 3,799 complete (91.5%) and 200 fragmented (4.9%) Benchmarking Universal Single-Copy Orthologs (BUSCOs). Only 105 BUSCOs (2.5%) were missing. Finally, according to Repeat Masker, 27% of both genomes had repetitive sequence, primary LINES (17% on both genomes) and LRT elements (4% on both genomes). Only Just 0.2 % were unclassified repeat elements ion both genomes (see Table S15 for details).

For the other species of SA canids, as well as additional (wild-caught) samples of bush dog and maned wolf, we extracted genomic DNA from 16 additional whole blood or tissue samples (Table S1) using the Qiagen DNEasy Blood and Tissue kit. Samples were chosen based on sufficient quality and quantity determined with a DNA fluorometer (Qubit 2.0), a NanoDrop spectrophotometer (ThermoFisher, USA), and gel electrophoresis. Genomic DNA samples were then used to prepare libraries with the Illumina TruSeq DNA Library Prep Kit, which were then 150 bp paired-end sequenced on an Illumina HiSeqX or NovaSeq 6000 instruments. Genomes from a culpeo fox and other canid species representing the genera *Canis*, *Cuon*, *Lupulella*, *Lycaon*, *Urocyon,* and *Vulpes* were downloaded from the National Center for Biotechnology Information's Sequence Read Archive (see Table S1 for further information).

**Mapping and genotype calling**

We filtered the raw reads using a modified pipeline from the Genome Analysis Toolkit (GATK) Best Practices recommendations (6, 7). Reads passing quality filters were mapped to the domestic dog CanFam3.1 assembly (8) using the Burrows-Wheeler Aligner with the MEM algorithm (9). We used GATK HaplotypeCaller to conduct combined genotype calling from sites that were mapped to the reference genome. We filtered the called genotypes for coverage and quality and kept only genotypes that had a minimum of 4 reads at a given position and Phred scores $\geq$20, and no more than the 99th percentile of coverage for each sample. Other variant filtering criteria followed the GATK Best Practices and (10). Briefly, we filtered out CpG islands, indels, multi-nucleotide polymorphisms, and sites with more than one alternate allele. The command line code used for read mapping, variant calling, and filtering is available at https://github.com/dechavezv/2nd.paper.v2

**Phylogenetic analysis**

We reconstructed a species tree representing the relationships among 31 genomes using ASTRAL-III (11). First, we mapped reads from 30 canid genomes to the domestic dog CanFam3.1 assembly. However, species tree estimation requires multi-species alignments in FASTA format. Therefore, we obtained from BAM files (individual genomes mapped to the domestic dog reference genomes) whole genomes in FASTA format. Consequently, we checked that our bam files contained only reads that had high quality (Phred scores >= 30), were in primary alignments, mapped in proper pairs, and were not PCR or optical duplicates. Specifically, we used the command `samtools view -hb -f 2 -F 256 -q 30 <Bam_File> | samtools view -hb -F 1024` (https://github.com/dechavezv/2nd.paper.v2/tree/main/). Then, we used bcftools (option mpileup and call -c) to conduct variant calling. Reads were filtered out for low-quality variants (q < 30 and Q <30), min depth coverage < 4x, and maximum depth coverage < 95th percentile of species total coverage). We converted the genotype calls to consensus FASTQ files using vcf2fq in vcfutils.pl from samtools and further transformed them into FASTA files with seqtk (https://github.com/lh3/seqtk/blob/master/seqtk.c). We used bedtools (12) to extract 6,716 regions, each 25 kb in length, from each of 31 canid genomes in FASTA format. Finally, we used PRANK v.150803 with one iteration (-F once option) to conduct a multi-species alignment on each of the 6,716 FASTA files (13, 14). The scripts used to extract the 25kb alignments from bam files can be found at https://github.com/dechavezv/2nd.paper.v2/tree/main/3-Phylogenomics/Write_25kb.

After trimming each of the 6,716 multiple sequence alignments with Gblocks (15), we calculated the gene tree phylogeny of each alignment using IQ-TREE 1 (16). We used 1000

ultrafast bootstraps and generated a consensus tree under the best model of substitution for each alignment using the modelFinder function (17). We then used ASTRAL-III v.5.5 (11) to infer a species tree while accounting for variation in the gene trees along the genome. We performed 100 bootstrap replicates and selected the best multi-locus tree based on maximum likelihood support values (Figure S1). We then scored the best tree to obtain posterior probabilities and quartet values for each node in the tree using ASTRAL-III v.5.5 and the quartet frequencies were visualized using a custom R script (see Figure S3). We used the red fox (*Vulpes vulpes*) as the outgroup to root the tree. The R script can be found at https://github.com/dechavezv/2nd.paper.v2/tree/main/3-Phylogenomics

**Average genomic divergence**

We calculated the average genomic divergence times from the 31 genomes analyzed in this study (including 18 new available genomes and the domestic dog reference genome). Specifically, we compiled 166,182 four-fold degenerate sites from 183 single-copy coding orthologs across these genomes. We used a custom python script (https://github.com/mahajrod/MAVR/tree/master/scripts/multiple_alignment/extract_degenerate_sites_from_codon_alignment.py) to concatenate these four-fold degenerate sites into a supermatrix. To estimate the substitution rate of this data matrix, we ran the BASEML program from the PAML 4.8 package (18) using the following settings in the BASEML control file (.ctl): runmode = 0 (user tree, i.e., the topology obtained from the ASTRAL-III analysis shown in Figure 1); model = 7 (REV-GTR); Mgene = 0 (rates); fix_kappa = 0; fix_alpha = 0; Malpha = 0 (one alpha); ncatG = 5 (number of substitution rate categories); fix_rho = 1 and rho = 0 (independent rates for sites); nparK = 0; clock = 1 (clock, rooted tree); nhomo = 1 (homogeneous

model for base frequency); getSE = 1 (obtain standard errors of estimates); RateAncestor = 0; cleandata = 0 (remove sites with ambiguity data = no).

We then used the supermatrix and the MCMCTree tool from the PAML 4.8 package (18), with the topology obtained from the ASTRAL-III analysis (Figure 1), to calculate divergence times. We employed the following settings in the MCMCTree control file (.ctl): ndata = 1 (one data partition); seqtype = 0 (nucleotides); usedata = 2 (use in.BV);  clock = 2 (independent rates); model = 4 (HKY + G model; (19); alpha = 0.5 (alpha shape parameter for gamma rates at sites); ncatG = 5 (number of substitution rate categories); cleandata = 0 (remove sites with ambiguity data = no); BDparas = 1 (birth) 1 (death) 0.1 (sampling); uniform node age priors generated; kappa_gamma = 6 2 (gamma prior for the substitution model parameters kappa); alpha_gamma = 1 1 (gamma shape parameter for variable rates among sites); sigma2_gamma = 1 10 1 (Dirichlet-gamma prior for the rate drift parameter); rgene_gamma = 2.000 2.000 (gamma prior for mean substitution rate); finetune = 1: 0.1 (times) 0.1 (rates) 0.1 (mixing) 0.1 (paras = parameters); 0.1 (RateParas = rate parameters) 0.1 (FossilErr); automatic finetuning of step size for proposals during the run of the MCMC. We ran the MCMC for 2,200,000 iterations, sampling every 2$^{nd}$ iteration, and discarding the first 200,000 iterations as burn-in. We estimated divergence times under the independent clock model with four fossil-based calibration priors (Table S16).

**Reconstruction of ancestral geographic areas**

We investigated the geographic origin of extant species of SA canids before possible colonization events across the Andes mountain chain using the R package BioGeoBEARS (20). This tool uses maximum likelihood to estimate the distribution of hypothetical ancestors (internal nodes) by modeling shifts between different geographical ranges along the phylogeny as a

function of time. We provided two input files: one containing a species tree (as inferred by ASTRAL-III), and the second containing information about whether a particular species is currently located in the west, east or central region of the Andes. We tested three different models of range evolution: dispersal-extinction-cladogenesis (DEC), dispersal vicariance analysis (DIVALIKE), and the Bayesian analysis of biogeography (BAYAREALIKE). Additionally, we tested the same three models plus a founder effect parameter on each model named "J" (for jump dispersal): DEC+J, DIVALIKE +J, and BAYAREALIKE+J (Figure S2) (20). The best-fitting model was chosen based on corrected Akaike information criterion (AICc) scores (Table S2). Among the six different models tested, the best-fit model included dispersal vicariance with a founder (jump dispersal) event (corrected AIC = 81.23%; Figure 1 and S2; Table S2).

**A multi-species demographic model with gene flow**

To obtain a detailed demographic model for SA canids, we applied G-PhoCS v1.3.2 (https://github.com/gphocs-dev/G-PhoCS) to 11,636 putatively neutral 1 kb windows, following (21) after mapping reads from 30 canid genomes to the domestic dog CanFam3.1 assembly. The analysis covered 16 genomes from the 14 species depicted in Figure 2, covering all ten SA canid species, as well as the coyote and gray wolf from North America, black-backed jackal, and a gray fox outgroup. For Darwin's fox and the SA gray fox, we used both sequenced genomes, because they represent different geographical regions. For the bush dog and maned wolf, we selected a single genome per species (bush dog 313 and maned wolf 370; Table S1). For the population phylogeny, we assumed the topology of the tree inferred with ASTRAL-III, and we augmented this tree with 104 total directed migration bands. These bands correspond to all 90

ordered pairs of sampled SA canid species, and 14 migration bands between lineages in the clade of bush dog and maned wolf and ancestral lineages in the clade of the remaining eight SA canids. Because of the large number of migration bands, we split the analysis into two separate runs. One run excluded the pampas fox genome and the 18 migration bands that involve it, and another run that included all genomes, but excludes the 28 migration bands between either bush dog or maned wolf and each of the other seven sampled SA canid species (including migration bands involving pampas fox).

For each run, we used a standard configuration for G-PhoCS, with the prior distribution of all the mutation-scaled population sizes ($\theta$) and divergence times ($\tau$) set to an exponential distribution with a mean of 0.0001, and the prior of all migration rates ($m$) set to a Gamma distribution with $\alpha$= 0.002, $\beta$= 0.00001. We ran a multi-threaded version of G-PhoCS with five threads per run and let the Markov chain Monte Carlo (MCMC) converge for 200,000 burn-in iterations, after which parameters were sampled every 50 iterations, for the next 400,000 iterations, resulting in a total of 8,000 samples from the approximate posterior distribution. For each parameter, we recorded the mean sampled value and the 95% Bayesian credible interval (CI). Population size estimates (Ne) were obtained from the mutation-scaled samples ($\theta$) by assuming a mutation rate per generation of $\mu$=4.0×10$^{-9}$ (22), and divergence times ($T$) were calibrated by assuming the same rate and an average generation time of four years based on the most current assessment of generation times for SA canids (23) (Table S17).

Migration probabilities were computed by the formula $prob$ = 1-$e^{-mt}$, where $m$ is the inferred migration rate and $t$ is the duration time of the migration band. This formula yields the probability that a lineage in the target population originated from the source population. The 28 migration bands that were included in the first run but not in the second run were inferred to have

migration probabilities below 0.1% (95% Bayesian CI). Thus, the results we show in Figure 2a, b and Table S3 are all derived from the second run, which includes all 14 species.

**Genomic diversity**

We examined the site heterozygosity in non-overlapping 100 kb windows across the genome of every SA canid analyzed. We defined heterozygosity as the number of heterozygous genotypes divided by the total number of sites that were called. The total genotypes called within each window included the sum of heterozygous, homozygote derived, and homozygote reference genotypes. We kept only windows with no more than 20% of missing data. The script used to calculate heterozygosity within 100 kb windows with a 10 kb step size was modified from (10) and is available at https://github.com/dechavezv/2nd.paper.v2/tree/main/4-Demography/Heterozygosity/WindowHet. We then quantified the extent of runs of homozygosity (ROH) in SA canids using PLINK (24). The parameters chosen to calculate ROH were SNPs within a window =200, heterozygotes allowed within a window= 3, and missing sites within a window=50. We binned these segments into three different size categories using PLINK (24; Table S4 and Figure 3d). The categories were short ROH [0 Mb - 1 Mb), medium ROH [1 Mb - 10 Mb), and long ROH [10 Mb - 100 Mb).

**MSMC**

We used the PSMC' model within the program MSMC (25) to calculate the instantaneous inverse coalescence rates (IICR) among SA canid lineages. We scaled the IICR from the MSMC model by 2μ to use it as a proxy of the effective population size (Ne). Following (26) IICR were further scaled using different inputs of generation time and mutation

rate to estimate plausible time ranges (in thousands of years) of the resulting Ne trajectories (Figure S7). We focused on species that inhabit forests, the bush dog and short-eared dog, and the savanna (i.e., Cerrado) specialist, the maned wolf since we predicted these species to have the most contrasting changes in Ne due to forest habitat contraction or expansion during Pleistocene climatic cycles (see Discussion for further details). Our range of mutation rates and generation times included a “low” mutation rate, which is the lowest extreme of our mutation rate interval, calculated assuming only a 1-year generation time. The “GW” mutation rate (darkest lines) was calculated based on the value used for the gray wolf in previous studies (27) and our G-PhoCS model with a 4-year generation time. The “reasonable” mutation rate (which is presented in the main text and Figure 4) was inferred based on the time of Ne decrease of bush dog and short-eared dog, which were the closest to the Last Glacial Maximum (LGM), 19-26.5 kya (28), when forest habitat was drastically reduced. The “high” mutation rate was the highest extreme on our mutation rate interval, calculated assuming a 10-year generation time.

**Positive Selection**

To obtain the orthologous genes necessary for implementing the branch-site model in Codeml, contained in the PAML 4.8 package (18), we followed (21). Briefly, we used BioMart in Ensembl (Ensembl 104 release) to obtain the coordinates of 39,704 transcripts belonging to 32,704 genes from the CanFam3.1 domestic dog genome. These regions were extracted from the annotation of the CanFam3.1 domestic dog reference genome. To avoid the inclusion of paralogous genes, we used VESPA (29) to extract and concatenate different exons from the same transcript. Also, sequences were filtered for quality (GQ≥30) and coverage (5< X <95 percentile). We further confirmed that exons had a permissible length (exons whose length is an

exact multiple of three) and contained no internal stop codons. We kept only the longest transcript for downstream analysis. Once sequences were translated into amino acid sequences, we aligned them with PRANK v.150803 (13) using the topology obtained by ASTRAL-III as a guide tree. The resulting multiple sequence alignments were reverse-translated to nucleotide sequence using VESPA (29). The pipeline to extract 1:1 orthologous genes from each species can be found at https://github.com/dechavezv/2nd.paper.v2/tree/main/2-PositiveSelection.

From the initial set of 32,704 genes (see above for details), we kept 17,181 genes as they represent the longest isoform, had no internal stop codons, and their gene length was a multiple of three. These genes were tested for signals of positive selection using the branch-site model in Codeml of the PAML 4.8 package (18). We compared a model that allows sites to be under positive selection (dN/dS > 1) along a particular branch in the tree (fix omega = 0) against a model where sites evolve under neutral or purifying selection (dN/dS = 1; fix omega = 1). To eliminate false positives from our scan of positively-selected sites, we masked regions with an overrepresentation of amino acid changes with SWAMP (30). Specifically, we masked any region with more than 10 amino acid changes in a 15-codon window, followed by 3 amino acid changes in a 5-codon window. Then, we visually inspected the gene alignments that had $p < 0.05$. We found that genes with extremely high likelihood ratios (LRT > 30) had nucleotide or amino acid differences caused by misalignments. After keeping only alignments that passed our filters, we conducted three independent runs for each foreground branch and gene and retained the one with the highest likelihood-ratio score of each run. Then, to mitigate a multi-nucleotide bias on our list of candidate genes (31), we identified genes with multiple nucleotide changes in a single codon that tended to increase the likelihood-ratio scores. Notably, these nucleotide

changes could occur simultaneously, but the branch-site model would interpret them as successive mutation events, thus inflating the likelihood scores. We found that none of the candidate genes reported in this study had multiple nucleotide changes in a single codon.

The likelihood of each model was compared through likelihood ratio tests (LRTs). We determined statistical significance using a chi-square distribution with 1 degree of freedom (32). Two forward branches were tested for selection, the bush dog clade (n = 4) and the maned wolf clade (n = 5) following the topology in Figure S1. We corrected for multiple hypotheses using a false discovery rate of 0.20 with QVALUE in R (33). Despite the considerable number of genes tested, we found only seven genes showing significant signals of positive selection (Q-value < 0.2). Finally, to avoid any potential reference bias in our positive selection results, we used the *de novo* genome assemblies of the bush dog and maned wolf and BLAST (34) to confirm candidate genes containing inferred positively-selected sites.

**Testing for polygenic selection**

We used polysel (35) to detect biological pathways overrepresented by weak to moderate signals of selection in the bush dog and maned wolf. We used the output from the branch-site model in the bush dog and maned wolf to find polygenic selection across biological pathways. We extracted these pathways from NCBI (https://www.ncbi.nlm.nih.gov/biosystems/) using the option "pathway"[BioSystemType] and "Canis lupus familiaris"[Organism]. Based on the literature, we chose pathways that are relevant to the unique morphologic features of the bush dog and maned wolf: diet (carnivorous or frugivorous), limb development, tooth formation, and interdigital membrane development. Polysel uses two inputs. One is the set of biological

pathways. The other is the gene set from PAML 4 in the form of 'SUMSTAT' scores. To obtain these scores, we took the fourth root of the log-likelihood ratios from the PAML 4 output. To make the ID of genes match those in the pathway, we converted gene labels into Entrez gene IDs using gene2ensembl from NCBI (ftp://ftp.ncbi.nih.gov/gene/DATA/gene2ensembl.gz). Polysel uses the genes and pathways to generate a null distribution. This null distribution was created by randomly sampling genes to make new pathways of a similar size. We obtained p-values by comparing the 'SUMSTAT' score between the null distribution and those from the original set. After correcting for multiple hypothesis testing as implemented in polysel, we chose significant pathways with an FDR <0.20.

**Enrichment of private alleles in gene flaking regions**

We aimed to detect enrichment of private alleles (alleles unique to one species) in the flanking regions of genes from the bush dog and maned wolf. First, we inferred interspecific variation by conducting joint genotyping of different canid species with HaplotypeCaller from GATK (6). This group included species of the genus *Chrysocyon* (maned wolf), *Speothos* (bush dog), *Lycalopex* (SA foxes), *Canis* (wolves, coyote, and golden jackal), *Cuon* (dhole), *Lycaon* (African wild dog), and *Lupulella* (African jackals). Then, we combined independent gVCF files with the "CombineGVCFs" option. We annotated the combined VCF for sites that passed our filter criteria (see the Mapping and genotype calling section for further details). We used BioMart in Ensembl (Ensembl 104 release) to extract the start and end sites from 39,704 transcripts belonging to 32,704 genes from the CanFam3.1 domestic dog genome. We then generated a .bed file with 1 kb windows upstream to the transcription start site (promoter region) and downstream from the transcription end site (potential regulatory region). We used this .bed

file to calculate the number of private alleles for the bush dog and maned wolf in the combined VCF file containing genotype calls for the different canid species. Within each 1 kb window, we used a custom Python script to calculate the number of private alleles for the bush dog and maned wolf. In parallel, we calculated the average number of variable sites across the remaining species (excluding maned wolf and bush dog). Considering that unique mutations at flanking regions could be overrepresented in the bush dog and maned wolf due to their relatively long evolutionary history (i.e. long branch lengths), we calculated the difference between private alleles between both species. Mutations overrepresented in one species but not in the other could be candidates for genes under positive selection. These estimates were calculated with the following formula (equation 1):

$$\frac{P}{S} = \frac{\frac{\sum_{i=1}^{L}(A_i + 0.5a_i) - (B_i + 0.5b_i)}{L}}{\frac{\frac{\sum_{i=1}^{L}X_i + 0.5Y_i}{n}}{L}}$$

where $A_i$ represents one private allele (allele unique to a particular species) in the bush dog and $a_i$ represents a private allele in a heterozygote state in the bush dog; notice that "$a_i$" is multiplied by 0.5 to account for heterozygosity. $B_i$ represents one private allele in the maned wolf genome and $b_i$ represents one private allele in a heterozygote state in the maned wolf; "$b_i$" is multiplied by 0.5 to account for heterozygosity. $X_i$ is the number of derived alleles in other canids (except bush dog and maned wolf) at a particular locus with respect to the domestic dog, and $Y$ is the number of heterozygous sites. This term is then divided by $n$, which is the number of samples analyzed. The sigma notation indicates that each private allele (numerator) or derived allele (denominator) will be added up to get a final sum of alleles within a particular window; "$i$" is

associated with a given locus/site. Then we normalized this value dividing it by the same term "*L*". After calculating the formula above on 38,542 1 kb windows, we calculated empirical *p*-values. Specifically, we filtered windows with less than 500 sites with data and then chose the value of P/S that indicated the top 1% of the remaining number of windows. To verify that the number of sites was not influencing outlier windows, we plotted P/S vs. the number of sites (Figure S8). Only windows with a minimum of 250 sites were included in the results. The custom pipeline can be found at https://github.com/dechavezv/2nd.paper.v2/tree/main/2-PositiveSelection/07-Selection_Regulatory_Regions/scripts.

## Supplementary Results

### Deleterious variation

To investigate the potential consequences of past population declines on SA canid species, we analyzed the mutational spectrum of protein-coding variants, particularly those that might be putatively deleterious. We assessed the effects of the joint variant calls from each of the 10 SA canid species on their associated protein-coding genes using the Variant Effect Predictor tool (36). First, we annotated mutations as synonymous or nonsynonymous. Then we used the SIFT scores to evaluate the effect of coding mutations on the associated protein. The SIFT score was calculated with SIFT (version 5.2.2) by comparing amino acid mutations annotated from the domestic dog reference genome with the UniRef90 protein database (release 2014_11). We grouped synonymous and tolerated mutations (SIFT score > 0.05) as 'benign' and classified loss-of-function mutations, deleterious missense mutations (SIFT score < 0.05), and variants that interrupt splice sites as 'damaging', following (10). Finally, we classified 'benign' and 'damaging' mutations as derived (not matching the reference allele) or ancestral (other canids

matching the reference allele) concerning the domestic dog genome. To avoid any potential reference bias in our results, we used BLAST (34) to confirm that the sequence of every candidate gene containing deleterious variants mapped to the *de novo* assemblies available for SA canids, specifically, the bush dog and maned wolf.

We found that the bush dog genomes had an average of 227 damaging homozygote derived genotypes, roughly 30% more than other SA canids, which averaged 165 (Figure S4 and Table S5). Notably, bush dogs averaged only 13% more benign homozygote-derived genotypes than the other species (Table S5). These findings suggest that the accumulation of deleterious variants in the bush dog could be a consequence of the long decline in effective population size. This result is consistent with observations that selection is inefficient in small populations, resulting in the fixation of deleterious variants of moderately and slightly negative effects on fitness (10, 37-39).

**Regulatory regions enriched with mutations associated with bone elongation in bush dogs and maned wolves**

To test if genes involved in endochondral bone elongation through chondrogenesis are uniquely associated with bush dog and maned wolf regulatory regions, we evaluated the proportion of private alleles in the regulatory regions of other canid species representing different clades across the species tree. These species included the culpeo fox, coyote, gray wolf, dhole, side-striped jackal, black-backed jackal, African wild dog, and Ethiopian wolf. Consistent with our expectations, we found that the bush dog has twice the number of signatures in limb-related genes with respect to other canids. Furthermore, only the bush dog and maned wolf had

positively selected genes related to bone elongation through chondrogenesis (Table S13).

## Supplementary Discussion

### Comparison between phylogenetic divergence and demographic divergence

G-PhoCS and MCMCtree represent two different methodological approaches to obtain estimates of species divergence times. Both methods assume a given species tree (here inferred by ASTRAL-III), but they employ a different set of assumptions in their models. MCMCtree employs a phylogenetic model, so its inferred times represent average times for common ancestry between individual lineages belonging to diverged species. Importantly, these time estimates do not consider the time period between this common ancestor and the divergence of the ancestral population to which it belonged. Thus, times inferred by MCMCtree are, by definition, upper bounds on the species divergence times. In cases where the ancestral populations are large and their divergence is relatively recent, as is the case with SA canids, the gap between species divergence and the common ancestor of individual lineages can be quite large. This issue and other limitations of Bayesian phylogenetic methods have been discussed in the literature, among others by the developers of MCMCtree (see eg., 40, 41).

Demography inference methods, such as G-PhoCS, are specifically designed to disentangle the time until coalescence in the ancestral population from the time to the most common recent ancestor. Thus, the divergence times they infer better reflect species divergence times. G-PhoCS also considers post-divergence gene flow, which is ignored by MCMCtree. Because gene flow increases the genetic similarity between diverged populations, it has an

opposite effect on estimates and will tend to reduce divergence times estimated by MCMCtree. Another technical, yet important, difference between the two sets of estimates is the method of calibration. MCMCtree uses priors defined by fossil data. Calibration of G-PhoCS estimates makes use of an assumed average mutation rate (per generation) and an average generation time. Ideally, one would want to use all sources of information in the calibration, but unfortunately, no existing method provides this. When considering the specific case of SA canids, which experienced recent divergence, have large ancestral population sizes, and have considerable levels of ancestral gene flow, we expect G-PhoCS to produce estimates that more accurately reflect species divergence times, when compared to MCMCtree.

When comparing the two sets of estimates we obtained from the two methods, we see that nearly all MCMCtree-based estimates are larger than their G-PhoCS-inferred counterparts, although with much larger confidence intervals (Table S18). This difference is fairly modest for the deep divergences, such as the basal divergence of SA canids (N24; inferred at 3.39 by MCMCtree and at 3.51 by G-PhoCS), Bush dog – Maned wolf (N14; inferred at 3.05 mya by MCMCtree and at 3.1 mya by G-PhoCS) and Short-eared dog – Crab-eating fox (N15; inferred at 2.11 mya by MCMCtree and at 1.87 mya by G-PhoCS) (Table S18). On the other hand, divergence times within the *Lycalopex* genus are inferred to be up to 70% higher by MCMCtree when compared to the corresponding times inferred by G-PhoCS (e.g., N21; inferred at 1.72 mya by MCMCtree and at 1.02 mya by G-PhoCS). This is largely due to the large ancestral effective sizes of the population directly ancestral to the *Lycalopex* clade, which is estimated at roughly 100,000 individuals. The average coalescence time in such a large population is expected to be roughly 100,000 generations, which is 0.4 million years (Table S18). The prevalent gene flow within this clade does not completely reverse this trend, and MCMCtree-based estimates are still

larger than those inferred by G-PhoCS (Table S18). However, the high rates of gene flow inferred for the pampas fox considerably reduce the gap between the divergence time inferred for it by MCMCtree (1.21 mya) and G-PhoCS (1.01 mya). This comparison confirms our prior expectation.

**Fossil record and the invasion of canids into South America**

Our findings of a single dispersal of canids into South America between 3.9 and 3.5 mya are consistent with previous molecular studies (42-44). This model has been challenged by the presence of North American fossils assigned to the maned wolf (*Chrysocyon*) and crab-eating fox (*Cerdocyon*) lineages from the early Pliocene, ~5 mya (45-47), suggesting that these groups predate the closure of the Panamanian land bridge (48, 49). However, these fossil remains are fragmentary, and correct phylogenetic assignments have proven difficult, precluding their use as conclusive evidence against a single-dispersal model. The North American *Cerdocyon* fossil has been related to the Asian raccoon dog, *Nyctereutes procyonoides* (45, 47, 50), although this species has never been recorded in the Americas (45, 51, 52). The remaining North American fossils, belonging to *Chrysocyon nearticus*, have been assigned to the maned wolf lineage based on dental features (45, 53). However, the dentition of these fossil taxa is common to species that are distantly related to SA canids, such as *Canis* and *Vulpes* (45). Despite the uncertainty about the identification of *Ch. nearticus,* these fossils possess the angular process that is characteristic of SA canids (45). The generalized dentition of *Ch. nearticus* suggests that it represents a basal lineage related to the ancestor of all SA canids, an inference that is compatible with the single-dispersal hypothesis.

**Conservation implication of the Darwin's fox genomic diversity**

Darwin's fox from Nahuelbuta National Park in Chile had low genome-wide heterozygosity and this was the only wild canid genome with a substantial proportion of long ROH (Figure 3b, d, and e), suggesting recent inbreeding during a severe population decline. This species inhabits the Valdivian forest, which extends from 35°S to 48°S latitude (54). During the LGM, ice sheets expanded from the Andes to the Pacific Coast. Glacial expansion likely restricted the Valdivian forest's southern limit to 41°S (55), which may have led to a decrease in population size (56). Although the effective population size trajectories inferred from our MSMC analyses likely reflect past climactic changes, the observed long ROHs in the Darwin's fox genome are likely the result of more recent inbreeding, resulting from human-driven habitat loss. Satellite images have shown that 33% of the native habitat of Darwin's fox has been lost due to deforestation (57), coinciding with its near elimination from the mainland (58, 59). Current estimates suggest that only 78 individuals remain in two relict populations in Nahuelbuta National Park and the Valdivian coastal range (58-61). These populations are isolated from each other and sensitive to direct human persecution and the introduction of domestic species (59, 60, 62), raising concerns about further erosion of genetic diversity. Captive breeding and habitat restoration are urgently needed to allow mainland populations to expand to parts of their previous geographic range and retain genetic viability. In contrast, Darwin's fox from Chiloé Island showed higher heterozygosity and smaller blocks of ROH, consistent with the larger census size (~500 adults) of this island refugial population that is considered less threatened than the mainland populations (61).

## Supplementary Figures

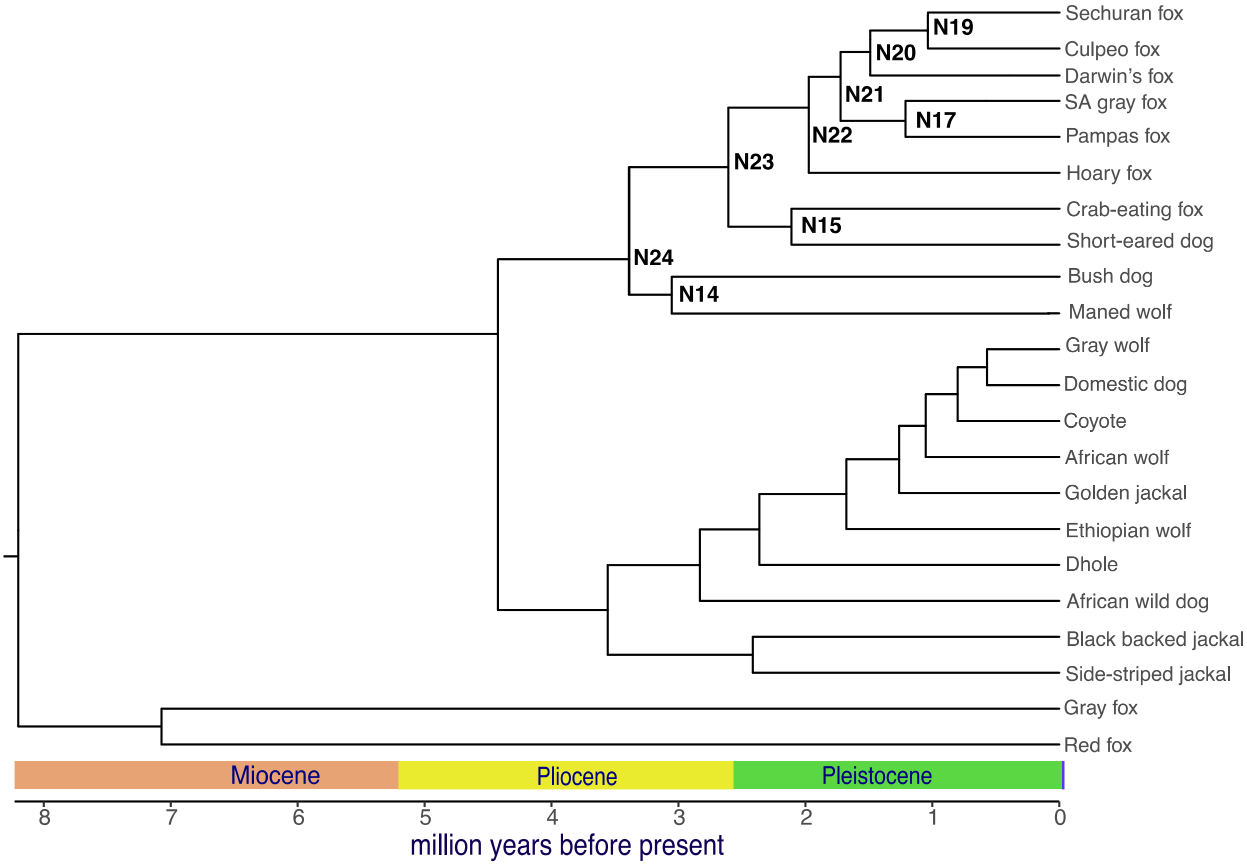


**Figure S1.** Consensus species tree based on 6,716 25 kb windows, as inferred by ASTRAL-III (11). A total of 31 genomes corresponding to 22 species are included in the tree (Table S1). Bootstrap support values (out of 100 replicates) are shown at the nodes of the tree. The divergence times were estimated using MCMCTree (18). See Table S18 for estimates with 95% credible intervals. A total of 31 genomes were included in the analysis, with multiple individuals used for bush dog, maned wolf, Darwin's fox, and SA gray fox. However, these have been collapsed in the presented tree.

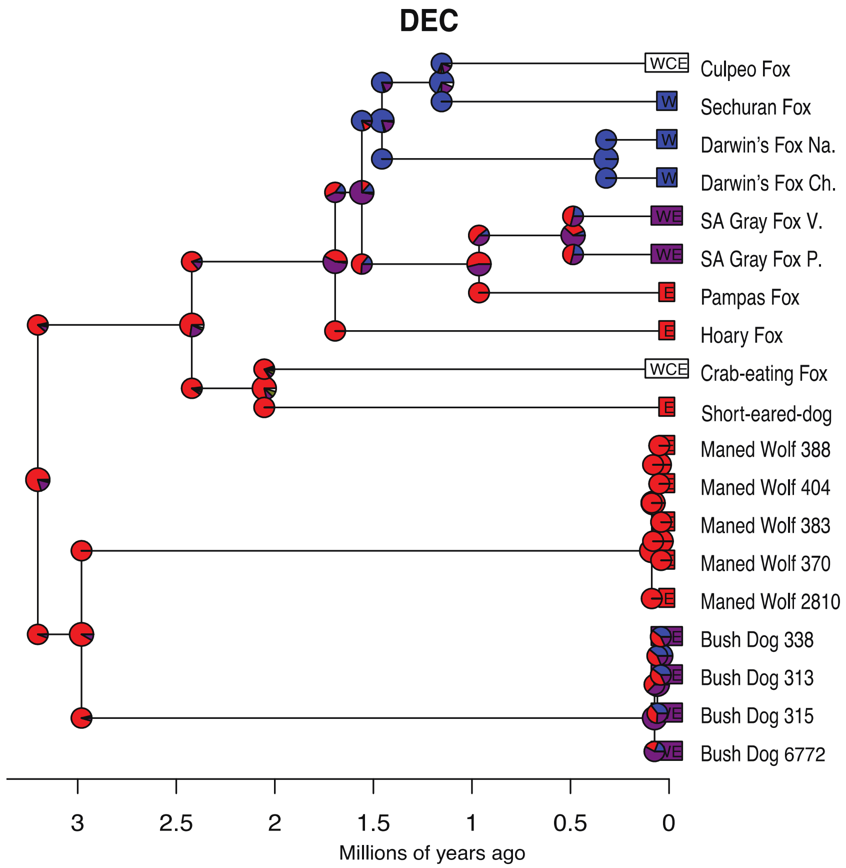
DEC
WCE
Culpeo Fox
W
Sechuran Fox
W
Darwin's Fox Na.
W
Darwin's Fox Ch.
WE
SA Gray Fox V.
WE
SA Gray Fox P.
E
Pampas Fox
E
Hoary Fox
WCE
Crab-eating Fox
E
Short-eared-dog
Maned Wolf 388
Maned Wolf 404
Maned Wolf 383
Maned Wolf 370
Maned Wolf 2810
Bush Dog 338
Bush Dog 313
Bush Dog 315
Bush Dog 6772
3
2.5
2
1.5
1
0.5
0
Millions of years ago

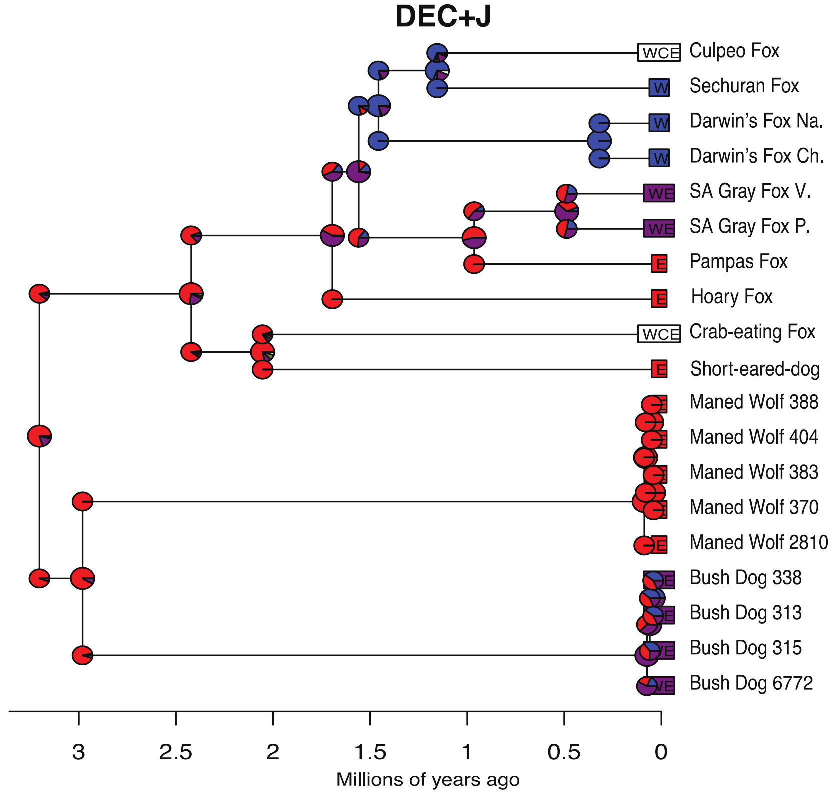
DEC+J
WCE
Culpeo Fox
W
Sechuran Fox
W
Darwin's Fox Na.
W
Darwin's Fox Ch.
WE
SA Gray Fox V.
WE
SA Gray Fox P.
E
Pampas Fox
E
Hoary Fox
WCE
Crab-eating Fox
E
Short-eared-dog
Maned Wolf 388
Maned Wolf 404
Maned Wolf 383
Maned Wolf 370
Maned Wolf 2810
Bush Dog 338
Bush Dog 313
Bush Dog 315
Bush Dog 6772
3
2.5
2
1.5
1
0.5
0
Millions of years ago

## DIVALIKE

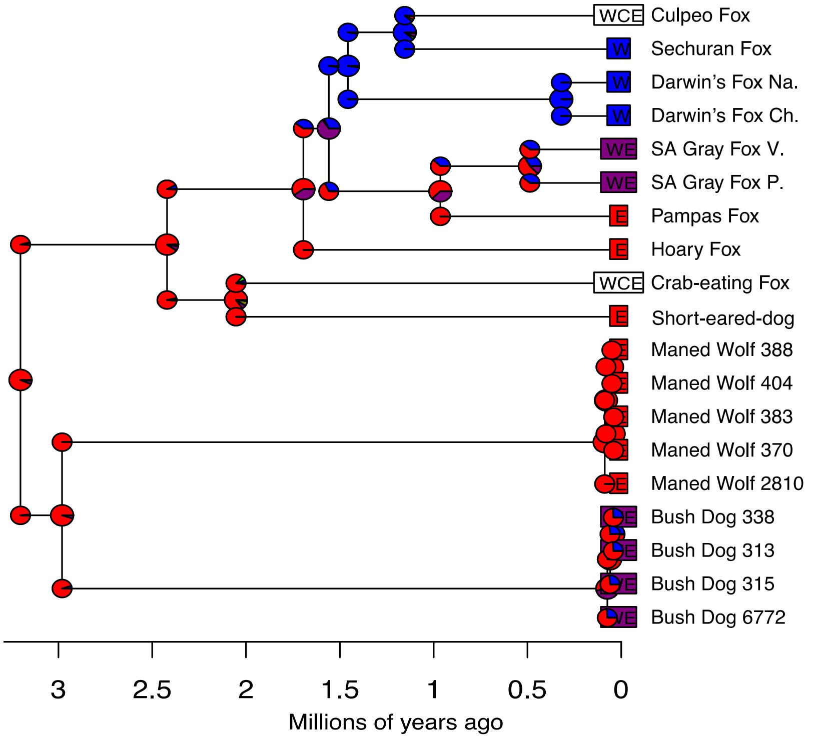


## DIVALIKE+J

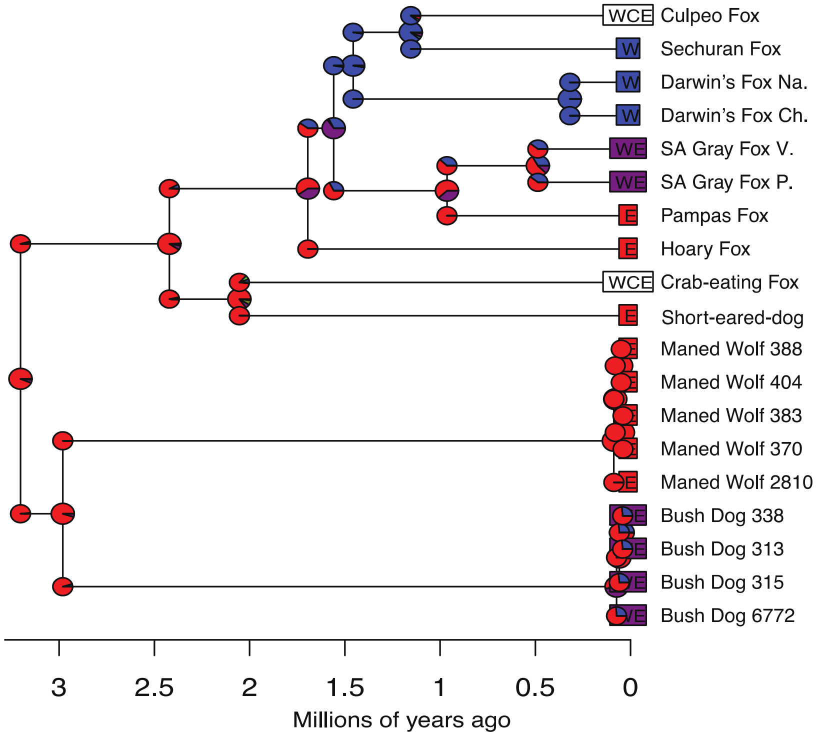

## BAYAREALIKE

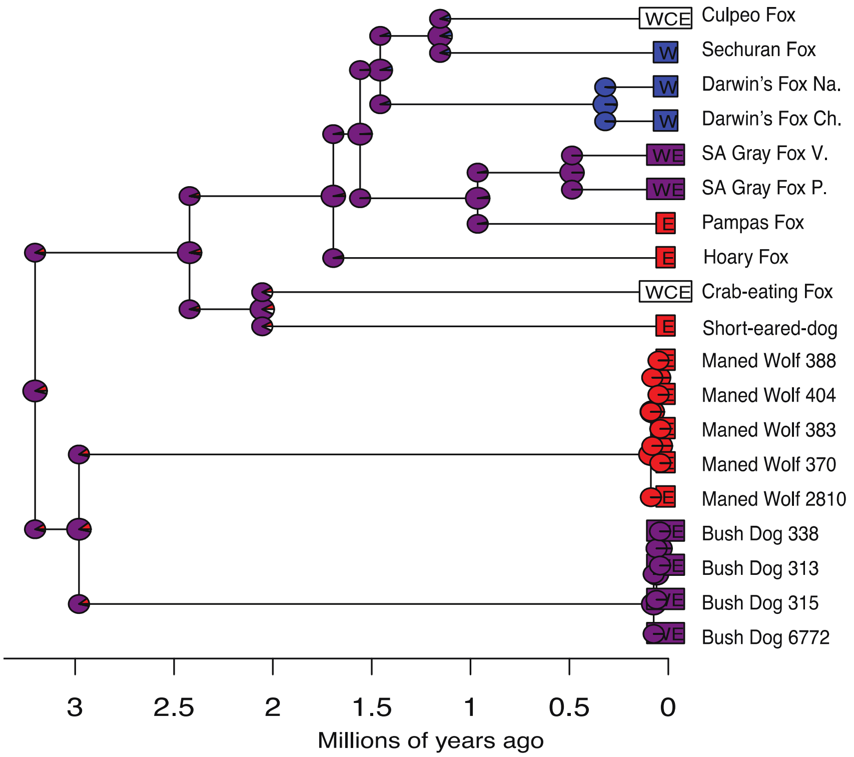


## BAYAREALIKE+J

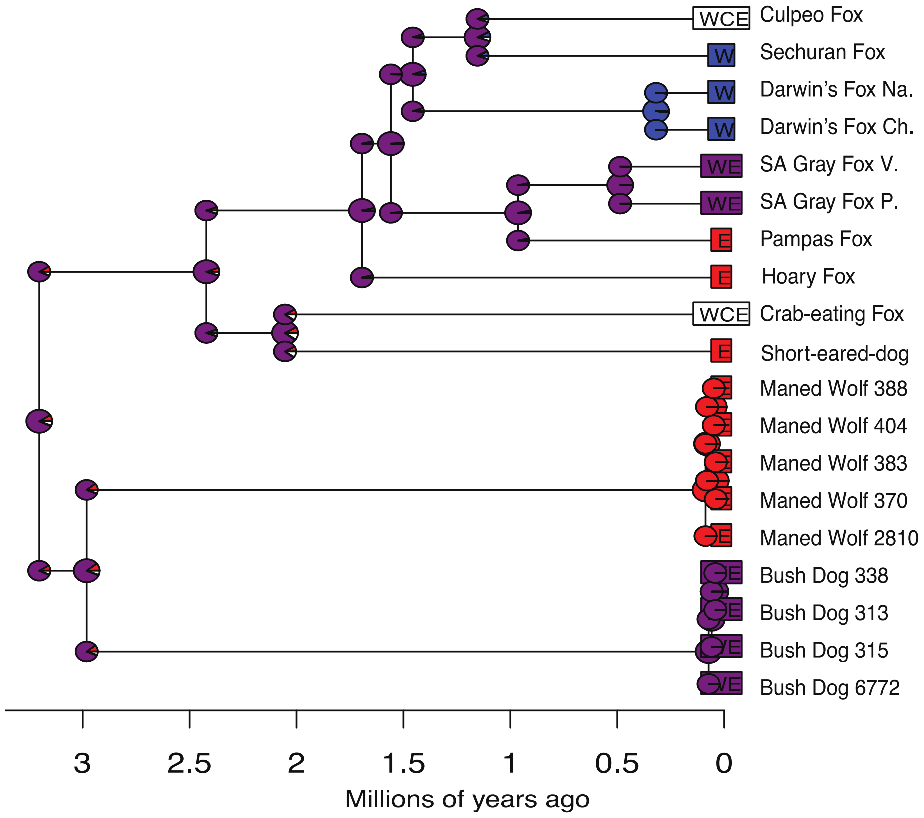

**Figure S2**. Inference of ancestral distributions inferred with BioGeoBEARS (20). Six different models were analyzed: DEC, DEC+J, BAYAREALIKE, BAYAREALIKE +J, DIVALIKE, and DIVALIKE+J. The parameter “J” represents a founder event. The probability of different ancestral distributions is indicated by the pie charts located at the nodes of the tree: red = East of the Andes, blue = West of the Andes, and purple = West and East of the Andes. Letters on terminal branches correspond to the species’ current distribution: W = west of the Andes, C = central region of the Andes, and E = east of the Andes. The model with the best corrected AIC score was DIVALIKE+J (see Table S2 for a full list of AIC scores).

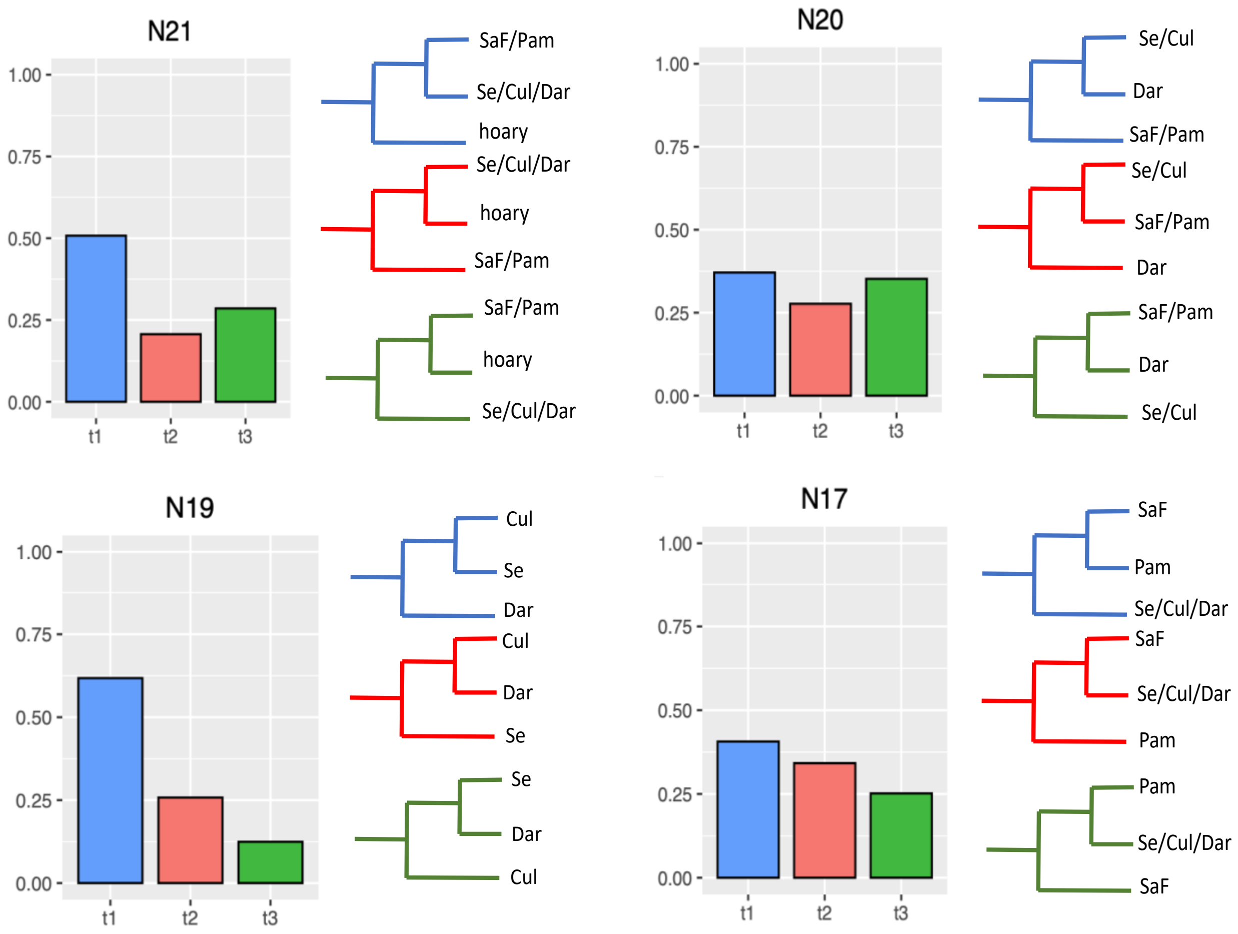


**Figure S3** Quartet frequencies for a subset of internal nodes of the species tree with three plausible tree topologies, as inferred by ASTRAL-III. The topology consistent with the inferred species tree in Figures 1 and 2 is shown in blue, and its frequency is compared with the frequencies of two alternative topologies (red and green). The labels on the terminal branches of the trees are Se = Sechuran fox, Dar = Darwin's fox, Cul= culpeo fox, Pam = pampas fox, SaF = South American gray fox.

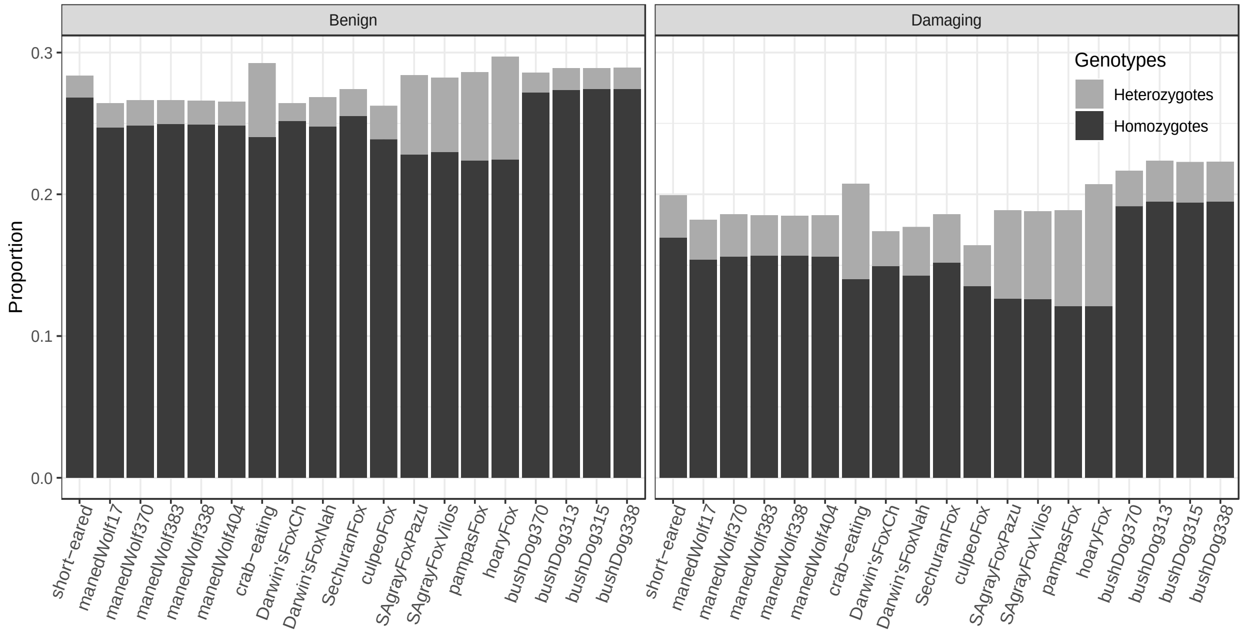


**Figure S4.** Proportion of derived alleles in the genome (ancestral and derived alleles). Mutations were identified as benign (synonymous and tolerated missense mutations) and damaging (deleterious missense mutations, disruption of splice sites, and gain or loss of a stop codon) using the Variant Effect Predictor tool (36). Only homozygous-derived genotypes and heterozygotes are shown. The full list of alleles, including homozygous-ancestral genotypes, is shown in Table S5. Homozygous-derived genotypes in the damaging category are higher in species with smaller estimated effective population sizes such as the bush dog and short-eared dog. In contrast, damaging mutations are less frequent in species with large effective population sizes, such as the hoary fox. This difference is less evident in the benign category.

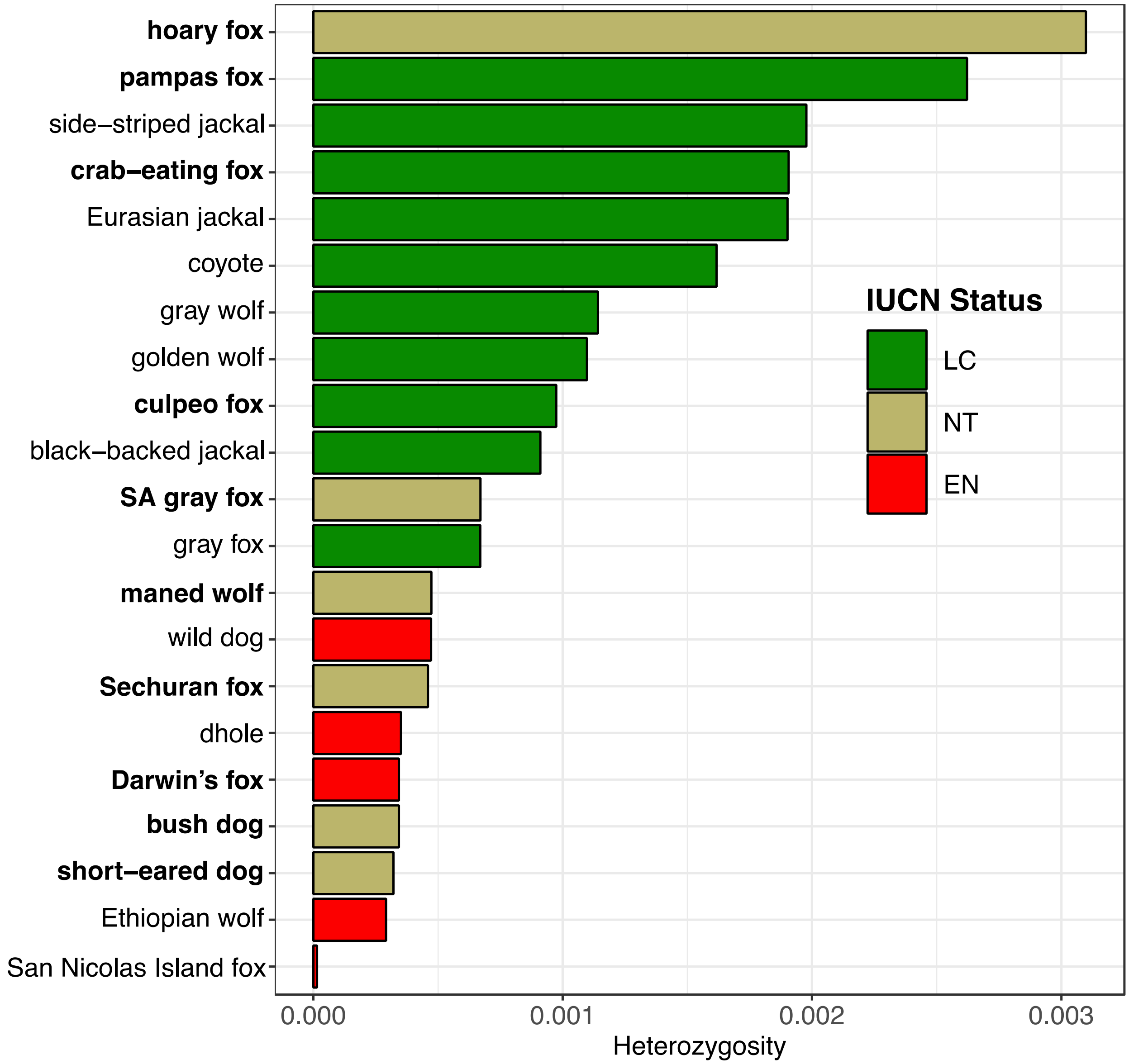


**Figure S5.** Genome-wide heterozygosity in different canid taxa. Names in bold indicate South American canids. The IUCN Red List categories are LC = Least Concern, NT = Near Threatened, and EN = Endangered. Endangered canids under the IUCN Red List have less than 0.5 heterozygote sites/kb. This group includes the African wild dog (wild dog), dhole, and Ethiopian wolf. South American canids with heterozygosity below this cutoff are the maned wolf, Sechuran fox, Darwin's fox, bush dog, and short-eared dog. Among these species, only Darwin's fox is listed as Endangered.

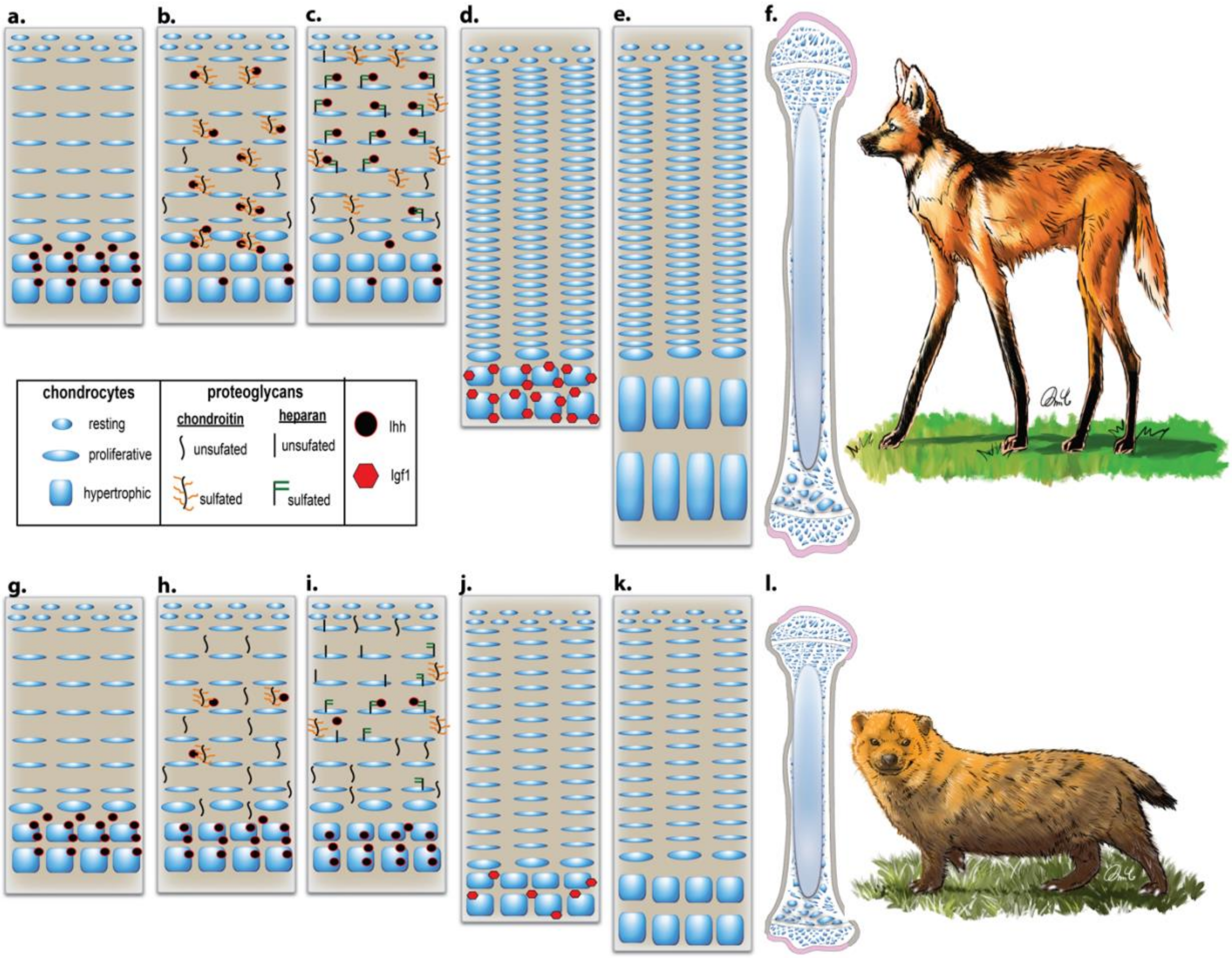


**Figure S6.** Limb elongation in the bush dog and maned wolf. The different panels represent the growth plate and the process of limb elongation in the endochondral bones of the maned wolf **(a-f)** and bush dog **(g-l)**. **(a and g)** Small circles at the top show the resting region, the middle-flattened circles represent the proliferative area, and the large squares at the bottom represent the hypertrophic region. The Indian hedgehog protein (Ihh; black circles) is initially produced by hypertrophic chondrocytes. Then, sulfated chondroitin proteoglycans transport Ihh to the top of the proliferative region, as shown in panels **(b and h)**. Note that unsulfated proteoglycans cannot transport Ihh. Panels **(c and i)** indicate the entrance of Ihh into the target cell facilitated by sulfated heparan proteoglycans. Note that Ihh will not ligate to unsulfated heparan. Once inside the cell, Ihh promotes the replication of chondrocytes in the proliferative region. Note the multiplication of chondrocytes in panels d and j. Finally, there is an enlargement of hypertrophic chondrocytes **(panels e and k)** regulated by the gene *IGF1***(d and j).** The rate of chondrocyte multiplication at the proliferative regions, coupled with hypertrophic enlargement, determines the length of the bone **(f and i).**

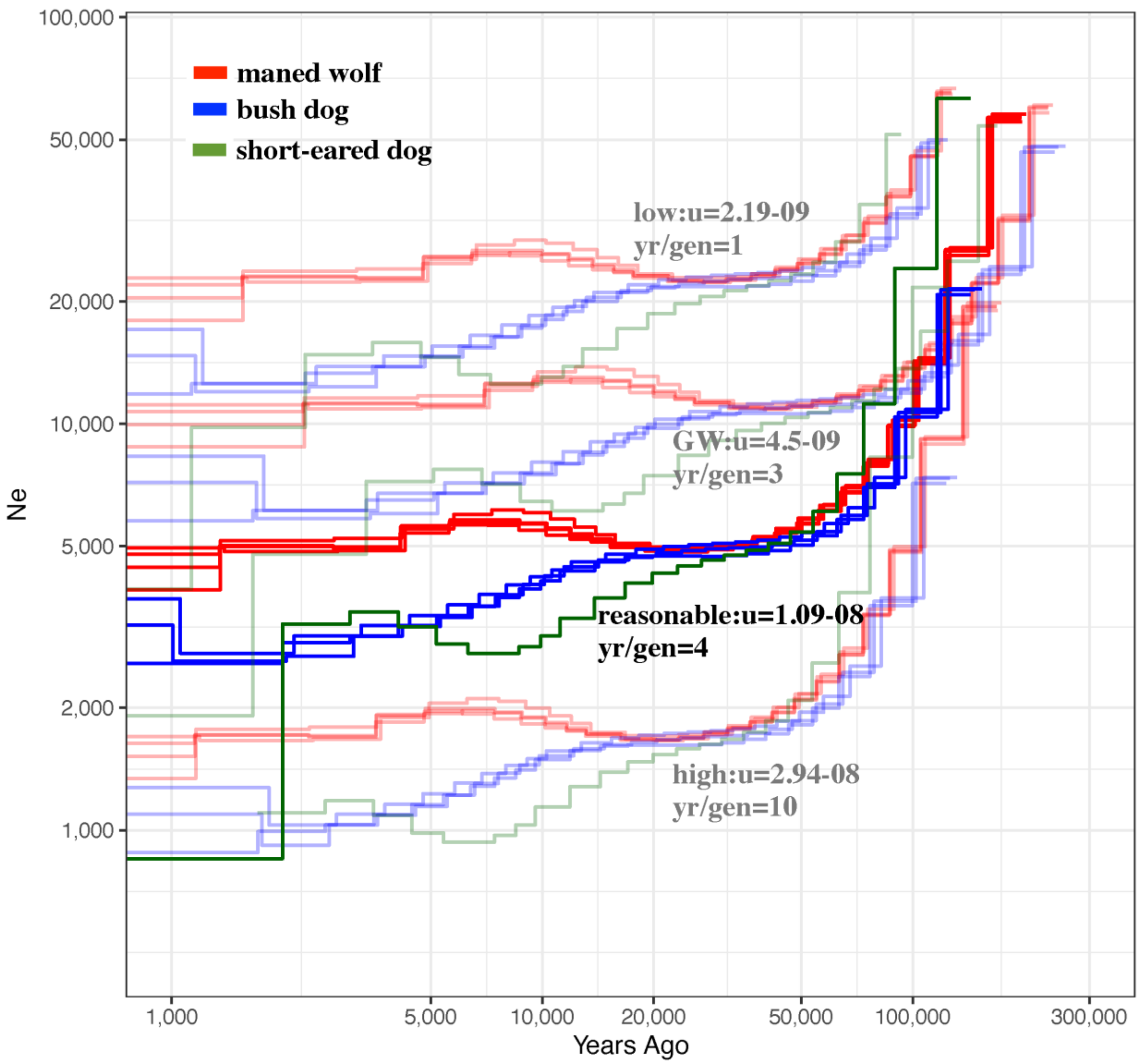


**Figure S7**. Demographic histories of the rainforest dwellers (bush dogs and short-eared dogs), and the savanna specialist (maned wolf) inferred using MSMC (25). Following (26), the figure shows the inference of inverse coalescent rates (IICR) scaled by 2μ, through time (in terms of years), for different paired values of mutation rate indicated by μ, and generation time shown as yr/gen. See Methods for details and justification for the different rates and generation times employed. Briefly, the "low" mutation rate (faint lines at the top) is the lowest extreme of the values tested. The "reasonable" mutation rate, which is presented in Figure 4 and the main text, is the closest to the LGM, 19-26.5 kya (28), thus a more plausible value (see Methods for details). The "GW" mutation rate (darkest lines) was used in our G-PhoCS model. The "high" mutation rate is the highest value among those tested. The range of mutation rates and generation times indicates how much the effective population size trajectories could vary with different assumptions for these parameters. For instance, the decline of the IICR in rainforest dwellers, the bush dog, and short-eared dog, could have started anywhere between 15,000 and 25,000 years ago, which is consistent with the Last Glacial Maximum (LGM), 19-26.5 kya (28).

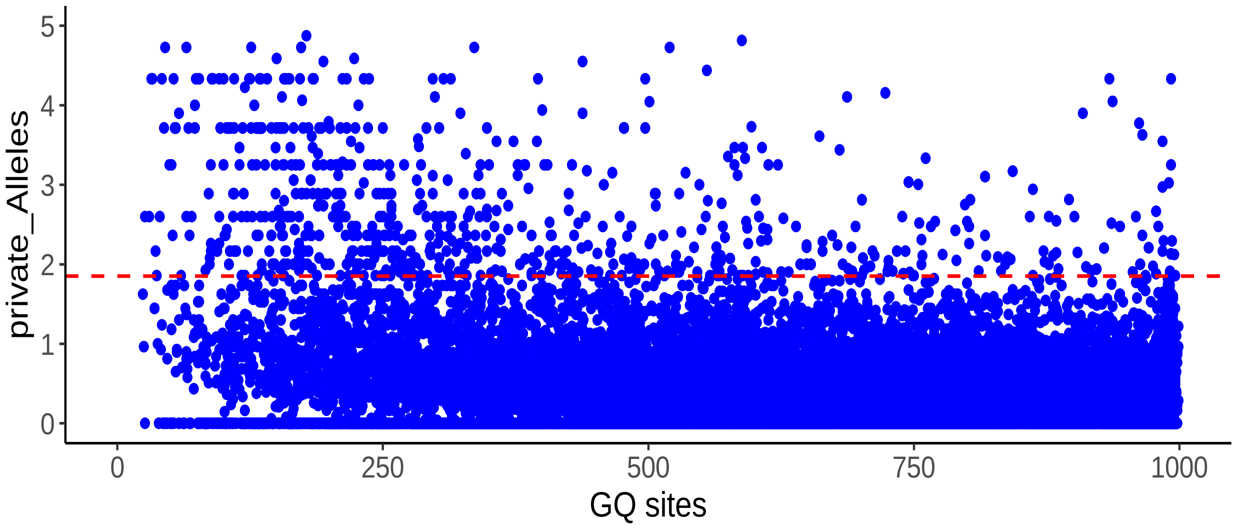


**Figure S8**. The relationship between private alleles and number of sites within 1 kb windows flanking 39,704 transcripts. The y-axis indicates the proportion of private alleles in the bush dog and maned wolf divided by the average number of segregating sites among other canids (see Methods for details). Significant genes (score equivalent to empirical p-value < 0.01) are shown above the horizontal red line. Specifically, we discarded windows with less than 500 sites and then chose the value of P/S that indicated the top 1% of the remaining number of windows. The x-axis indicates the number of sites within a particular 1kb window that passed our filter criteria (see Methods for details). The windows that contained less than 250 good quality sites ("GQ sites") were ignored.

## Supplementary Tables

**Table S1.** Descriptive statistics of the 30 canid genomes analyzed in this study (not including the CanFam3.1 domestic dog reference genome).

| **Common name** | **Scientific name** | **Sample ID** | **Reference** | **Mean depth coverage (X)** | **Base Pairs (Gbp)** | **% of reference covered by at least 3 reads** |
|---|---|---|---|---|---|---|
| Maned wolf 2810 | *Chrysocyon brachyurus* | Cbr17082018* | This study | 77.7 | 98.34 | 98.48 |
| Maned wolf 370 | *Chrysocyon brachyurus* | bcbr370 | This study | 27,01 | 62.9 | 98.29 |
| Maned wolf 383 | *Chrysocyon brachyurus* | bcbr383 | This study | 27.96 | 65 | 98.37 |
| Maned wolf 388 | *Chrysocyon brachyurus* | bcbr388 | This study | 22.1 | 51.4 | 98.23 |
| Maned wolf 404 | *Chrysocyon brachyurus* | bcbr404 | This study | 24.35 | 56.7 | 98.26 |
| Bush dog 16 | *Speothos venaticus* | Sve16082018* | This study | 42.4 | 98.8 | 98.5 |
| Bush dog 313 | *Speothos venaticus* | bsve313 | This study | 63.5 | 95.2 | 98.55 |
| Bush dog 315 | *Speothos venaticus* | bsve215 | This study | 20.37 | 47.4 | 98.15 |
| Bush dog 338 | *Speothos venaticus* | bve338 | This study | 24.7 | 57.5 | 98.22 |
| Short-eared dog | *Atelocynus microtis* | AMI-1 | This study | 17.23 | 40.1 | 98.23 |
| Crab-eating fox | *Cerdocyon thous* | bCth-213 | This study | 23 | 65.3 | 98.67 |
| Hoary fox | *Lycalopex vetulus* | bPvet-20 | This study | 32.12 | 74.8 | 98.72 |
| Pampas fox | *Lycalopex gymnocercus* | bPgy-20 | This study | 11.78 | 27.4 | 98.03 |
| SA gray fox Pazucar | *Lycalopex griseus* | Lgr0015 | This study | 16.7 | 38.6 | 98.4 |
| SA gray fox Vilos | *Lycalopex griseus* | Lgr006 | This study | 18 | 41.7 | 98.45 |
| Darwin's fox Chiloe | *Lycalopex fulvipes* | Dfu_Chiloe | This study | 16.1 | 37.4 | 98.25 |
| Darwin's fox Nahuelbuta | *Lycalopex fulvipes* | Dfu_Nah | This study | 19.3 | 45 | 98.4 |
| Sechuran fox | *Lycalopex sechurae* | DSE-2 | This study | 16.14 | 37.6 | 98.28 |
| Black back jackal | *Lupulella mesomelas* | BBJ | (63) | 57 | 132 | 98.9 |
| Side stripe jackal | *Lupulella adusta* | SSJ | (63) | 37.8 | 88 | 97.63 |
| African wolf | *Canis lupaster* | RKW1356 | (64) | 27.96 | 65 | 98.11 |
| Andean fox | *Lycalopex culpaeus* | SRS523207 | (65) | 10.98 | 25.5 | 97.71 |
| AWD South Africa | *Lycaon pictus* | SAMN09924608 | (21) | 27.93 | 65 | 98.71 |
| Coyote | *Canis latrans* | RKW13455 | (27) | 25.67 | 59.7 | 98.72 |
| Dhole | *Cuon alpinus* | SAMN10180424 | (66) | 19.53 | 45.4 | 98.35 |
| Ethiopian wolf | *Canis simensis* | SAMN10180425 | (66) | 9.66 | 22.5 | 97.37 |
| Golden jackal | *Canis aureus* | SAMN03366713 (RKW1332) | (67) | 26.09 | 60.7 | 97.46 |
| Gray fox | *Urocyon cinereoargenteus* | SAMN04495241 | (37) | 18.47 | 42.9 | 97.49 |
| Gray wolf | *Canis lupus* | RKW1547 | (68) | 27.33 | 63.6 | 98.76 |
| Red fox | *Vulpes vulpes* | SAMN06553695 | (69) | 70 | 173.2 | 98.60 |

Note - Statistics were calculated with Qualimap (70) on BAM files that were mapped to the CanFam3.1 domestic dog genome assembly (see Methods).
* Samples used for the construction of de novo genome assemblies.

**Table S2.** Descriptive statistics of the six ancestral species distribution models implemented using BioGeoBEARS (20).

| Model | LnL | #Parameters | d | e | j | AICc |
|---|---|---|---|---|---|---|
| DEC | -33.16 | 2 | 0.26 | 2.00E-08 | 0 | 71.06 |
| DEC+J | -33.16 | 3 | 0.26 | 1.00E-12 | 1.00E-05 | 73.91 |
| DIVALIKE | -36.81 | 2 | 0.3 | 1.00E-12 | 0 | 78.38 |
| **DIVALIKE+J** | **-36.81** | **3** | **0.3** | **1.00E-12** | **1.00E-05** | **81.23** |
| BAYAREALIKE | -19.71 | 2 | 0.071 | 0.14 | 0 | 44.16 |
| BAYAREALIKE+J | -19.58 | 3 | 0.08 | 0.18 | 1.00E-05 | 46.77 |

Note - The log-likelihoods for different models are shown in column "LnL". Each model contained a specific number of parameters. The best-fitting model (shown in bold) was selected based on the corrected Akaike information criterion (AICc) scores. The parameters are d = rate of range-expansion dispersal, e = rate of range-contraction/local extirpation, and j=founder event.

**Table S3.** Demographic parameter estimates inferred by G-PhoCS (https://github.com/gphocs-dev/G-PhoCS).

| Parameter | Mean estimate (95% Bayesian CI) |
|---|---|
| **Population divergence times (years)** | |
| **tau_N19** | 809800 (780700-836000) |
| **tau_N20** | 1010400 (983900-1037500) |
| **tau_N17** | 1012200 (985100-1039000) |
| **tau_N21** | 1013300 (986200-1040000) |
| **tau_N22** | 1397300 (1360400-1435500) |
| **tau_N15** | 1866600 (1808000-1922800) |
| **tau_N23** | 2246200 (2226700-2266400) |
| **tau_N14** | 3097100 (3049800-3146100) |
| **tau_N24** | 3515900 (3497700-3533700) |
| **tau_N1** | 269800 (256700-282600) |
| **tau_N29** | 2649400 (2611000-2687300) |
| **tau_N26** | 3883500 (3863600-3903300) |
| **tau_ROOT** | 9326500 (9232900-9425900) |
| **Effective population sizes (number of individuals)** | |
| **theta_SECHURAN_FOX** | 8700(8400-9000) |
| **theta_CULPEO_FOX** | 13800(13300-14400) |
| **theta_DARWIN'S_FOX** | 5300(5100-5500) |
| **theta_SOUTH_AMER._GRAY_FOX** | 19200(18500-19900) |
| **theta_PAMPAS_FOX** | 118400(114200-122600) |
| **theta_HOARY_FOX** | 52000(49500-54600) |
| **theta_CRAB-EATING_FOX** | 43000(41900-44200) |
| **theta_SHORT-EARED_DOG** | 6500(6200-6800) |
| **theta_BUSH_DOG** | 7900(7600-8200) |
| **theta_MANED_WOLF** | 12000(11600-12400) |
| **theta_GRAY_WOLF** | 21300(20200-22400) |
| **theta_COYOTE** | 36500(34400-38700) |
| **theta_BLACK-BACKED_JACKAL** | 22800(22200-23400) |
| **theta_GRAY_FOX** | 37300(36400-38200) |
| **theta_N19** | 22200(18100-26500) |
| **theta_N20** | 6200(700-15100) |
| **theta_N21** | 142000(130500-154500) |
| **theta_N22** | 105200(101300-109200) |
| **theta_N15** | 77300(65300-89600) |
| **theta_N23** | 62800(60900-64800) |
| **theta_N14** | 42800(35200-50800) |
| **theta_N24** | 11600(10900-12400) |
| **theta_N1** | 52600(51400-53700) |
| **theta_N29** | 80800(76700-85000) |
| **theta_N26** | 51400(50200-52600) |
| **theta_ROOT** | 81100(75700-86400) |
| **Migration probabilities (%)** | |
| **m_PAMPAS_FOX->SOUTH_AMER._GRAY_FOX** | 89.0(87.9-89.9) |
| **m_PAMPAS_FOX->DARWIN'S_FOX** | 63.2(61.1-64.9) |
| **m_PAMPAS_FOX->HOARY_FOX** | 51.2(48.2-53.9) |
| **m_CULPEO_FOX->DARWIN'S_FOX** | 13.6(12.2-14.9) |
| **m_PAMPAS_FOX->CULPEO_FOX** | 13.2(11.3-15.1) |

| | |
|---|---|
| **m_CULPEO_FOX->SOUTH_AMER._GRAY_FOX** | 11.6(10.4-12.8) |
| **m_DARWIN'S_FOX->SOUTH_AMER._GRAY_FOX** | 11.0(9.4-12.5) |
| **m_HOARY_FOX->SOUTH_AMER._GRAY_FOX** | 8.7(6.1-11.0) |
| **m_CULPEO_FOX->PAMPAS_FOX** | 5.6(4.7-6.3) |
| **m_HOARY_FOX->CRAB-EATING_FOX** | 5.4(4.4-6.5) |
| **m_N23->MANED_WOLF** | 5.2(3.7-6.6) |
| **m_DARWIN'S_FOX->HOARY_FOX** | 2.9(1.8-3.9) |
| **m_CULPEO_FOX->HOARY_FOX** | 2.8(2.1-3.6) |
| **m_PAMPAS_FOX->CRAB-EATING_FOX** | 2.3(1.5-3.1) |
| **m_HOARY_FOX->PAMPAS_FOX** | 2.2(0.0-4.1) |
| **m_CRAB-EATING_FOX->SHORT-EARED_DOG** | 1.7(0.9-2.6) |
| **m_SECHURAN_FOX->CULPEO_FOX** | 1.7(1.0-2.4) |
| **m_CULPEO_FOX->SECHURAN_FOX** | 1.1(0.5-1.8) |
| **m_PAMPAS_FOX->SECHURAN_FOX** | 1.0(0.2-1.8) |
| **m_CRAB-EATING_FOX->PAMPAS_FOX** | 0.9(0.7-1.1) |
| **m_SECHURAN_FOX->PAMPAS_FOX** | 0.7(0.4-1.1) |
| **m_SECHURAN_FOX->DARWIN'S_FOX** | 0.7(0.3-1.2) |
| **m_SHORT_EARED_D->CRAB_EATING_FOX** | 0.7(0.3-1.1) |
| **m_HOARY_FOX->DARWIN'S_FOX** | 0.7(0.0-3.3) |
| **m_PAMPAS_FOX->SHORT-EARED_DOG** | 0.7(0.4-1.0) |
| **m_SECHURAN_FOX->HOARY_FOX** | 0.5(0.2-0.9) |
| **m_N23->BUSH_DOG** | 0.4(0.0-2.1) |
| **m_N22->BUSH_DOG** | 0.4(0.0-0.8) |
| **m_DARWIN'S_FOX->CULPEO_FOX** | 0.4(0.0-1.3) |
| **m_CRAB-EATING_FOX->SECHURAN_FOX** | 0.4(0.2-0.6) |
| **m_SECHURAN_FOX->SOUTH_AMER._GRAY_FOX** | 0.3(0.0-0.8) |
| **m_HOARY_FOX->SECHURAN_FOX** | 0.3(0.0-0.8) |
| **m_BUSH_DOG->N23** | 0.3(0.1-0.5) |
| **m_N23->BD_MANED_WOLF** | 0.1(0.0-0.6) |
| **m_SOUTH_AMER._GRAY_FOX->DARWINS_FOX** | 0.1(0.0-0.5) |
| **m_SOUTH_AMER._GRAY_FOX->HOARY_FOX** | 0.0(0.0-0.5) |
| **m_HOARY_FOX->CULPEO_FOX** | 0.0(0.0-0.5) |

Note – The analysis was conducted under a model using a population phylogeny with the topology of the species tree inferred by ASTRAL-III (Figure 1) with 76 migration bands. Ancestral populations are indicated by the node labels (e.g N21), which match the nodes shown in Figures 1 and 2a. Effective population sizes were calibrated by assuming an average per-site mutation rate of $\mu=4.0\times10^{-9}$ (22), and divergence times were calibrated assuming the same rate and an average generation time of four years. Migration probabilities were computed by the formula $prob = 1-e^{-mt}$, where $m$ is the inferred migration rate and $t$ is the duration time of the migration band. This formula yields the probability that a lineage in the target population originated from the source population. Migration bands are sorted according to their inferred mean probability; migration probabilities for the 39 migration bands not shown here all had mean values below 0.1% and a 95% Bayesian CI below 0.5%. The same goes for 28 additional migration bands involving the bush dog and maned wolf, which were considered in a separate run of G-PhoCS (see Methods).

**Table S4.** Genome-wide average heterozygosity (per kb) and total length of short/medium/long runs of homozygosity (ROH) of South American canids.

| Genome | Heterozygosity (kb) | Short ROH (Mb) | Medium ROH (Mb) | Long ROH (Mb) |
|---|---|---|---|---|
| Hoary fox | 2.90 | 57.23 | 37.38 | 0 |
| Crab-eating fox | 1.78 | 119.98 | 128.38 | 0 |
| Pampas fox | 2.47 | 143.29 | 144.21 | 0 |
| SA gray fox los Vilos | 2.22 | 272.99 | 131.60 | 11.67 |
| Culpeo fox | 0.90 | 244.43 | 95.933 | 0 |
| SA gray fox Pan de azucar | 2.11 | 375.24 | 95.48 | 0 |
| Maned wolf 2810 | 0.47 | 368.17 | 36.82 | 26.30 |
| Maned wolf 388 | 0.47 | 404.38 | 47.55 | 11.63 |
| Maned wolf 383 | 0.48 | 405.86 | 64.19 | 25.71 |
| Sechuran fox | 0.49 | 424.55 | 75.77 | 0 |
| Maned wolf 370 | 0.49 | 411.28 | 47.75 | 0 |
| Maned wolf 404 | 0.47 | 428.43 | 50.79 | 0 |
| Darwin's fox Ch | 0.35 | 793.13 | 935.60 | 0 |
| Bush dog 6772 | 0.33 | 515.74 | 150.12 | 174.87 |
| Darwin's fox Na | 0.68 | 759.00 | 293.99 | 131.03 |
| Bush dog 313 | 0.36 | 647.203 | 73.28 | 0 |
| Bush dog 315 | 0.34 | 652.35 | 78.75 | 17.60 |
| Short-Eared-dog | 0.34 | 721.51 | 235.20 | 15.09 |
| Bush dog 338 | 0.35 | 663.25 | 81.12 | 0 |

Note - ROH were categorized as short (<1Mb), medium (1-10Mb), and long (>10Mb).

**Table S5.** Genotype counts in South American canids.

| Sampled | HomAnc | HomDerv | Het | MutType |
|---|---|---|---|---|
| short-eared dog | 8901 | 3333 | 194 | benign |
| Maned wolf 18 | 9230 | 3098 | 216 | benign |
| Maned wolf 370 | 9148 | 3098 | 223 | benign |
| Maned wolf 383 | 9071 | 3082 | 210 | benign |
| Maned wolf 388 | 9133 | 3096 | 212 | benign |
| Maned wolf 404 | 9156 | 3093 | 212 | benign |
| crab-eating fox | 8794 | 2986 | 651 | benign |
| Darwin's Chiloe | 9165 | 3132 | 158 | benign |
| Darwin's Nahuelbuta | 9092 | 3077 | 258 | benign |
| Sechuran fox | 9005 | 3164 | 241 | benign |
| Culpeo fox | 8627 | 2793 | 278 | benign |
| SA gray fox Vilos | 8931 | 2842 | 701 | benign |
| SA gray fox Pazucar | 8948 | 2863 | 653 | benign |
| Pampas fox | 8470 | 2653 | 744 | benign |
| Hoary fox | 8777 | 2804 | 908 | benign |
| Bush dog 16 | 8916 | 3392 | 174 | benign |
| Bush dog 313 | 8856 | 3407 | 192 | benign |
| Bush dog 315 | 8839 | 3406 | 188 | benign |
| Bush dog 338 | 8833 | 3407 | 187 | benign |
| short-eared dog | 940 | 199 | 35 | damaging |
| Maned wolf 18 | 974 | 183 | 34 | damaging |
| Maned wolf 370 | 959 | 184 | 35 | damaging |
| Maned wolf 383 | 949 | 182 | 34 | damaging |
| Maned wolf 388 | 956 | 184 | 33 | damaging |
| Maned wolf 404 | 960 | 184 | 34 | damaging |
| crab-eating | 927 | 164 | 78 | damaging |
| Darwin's Chiloe | 976 | 176 | 29 | damaging |
| Darwin's Nahuelbuta | 968 | 167 | 40 | damaging |
| Sechuran fox | 960 | 179 | 41 | damaging |
| Culpeo fox | 843 | 136 | 30 | damaging |
| SA gray fox Vilos | 964 | 150 | 74 | damaging |
| SA gray fox Pazucar | 964 | 150 | 73 | damaging |
| Pampas fox | 910 | 136 | 76 | damaging |
| Hoary fox | 936 | 143 | 102 | damaging |
| **Bushdog 16** | **929** | **227** | **30** | **damaging** |
| **Bush dog 313** | **912** | **228** | **34** | **damaging** |
| **Bush dog 315** | **913** | **228** | **34** | **damaging** |
| **Bush dog 338** | **909** | **228** | **33** | **damaging** |

Note – Homozygote-derived genotypes in the damaging category are higher in species with smaller effective populations sizes such as the bush dog (bold) due to increased drift. In contrast, damaging mutations are less frequent in species with large effective populations sizes such as the hoary fox. This difference is less evident in the benign category. The counts of benign and damaging alleles in the genome were 17,647 and 2,838, respectively.

**Table S6.** Candidate genes containing sites with signals of positive selection in the bush dog, as identified by the branch-site test in PAML 4.8 (18).

| **Ensemble_ID** | GeneSymbol | Pvalue | Qvalue |
|---|---|---|---|
| ENSCAFG00000019513 | MAPK8IP3 | 5.71E-07 | 0.00631248 |
| ENSCAFG00000003587 | TRAF1 | 5.93E-06 | 0.02536113 |
| ENSCAFG00000000814 | HLA-DQB2 | 1.85E-05 | 0.0632959 |
| ENSCAFG00000028925 | NDUFAF8 | 3.20E-05 | 0.09123733 |
| ENSCAFG00000015765 | TFE3 | 5.41E-05 | 0.13221267 |
| ENSCAFG00000019737 | KCNT1 | 8.02E-05 | 0.17149768 |
| ENSCAFG00000003181 | N/A | 0.00010989 | 0.20887647 |
| ENSCAFG00000017618 | DRP2 | 0.00024554 | 0.38185934 |
| ENSCAFG00000008863 | SCAF4 | 0.00027261 | 0.38862827 |
| ENSCAFG00000030681 | N/A | 0.00036163 | 0.47587726 |
| ENSCAFG00000003434 | VAX2 | 0.00053129 | 0.57534564 |
| ENSCAFG00000030605 | N/A | 0.00053629 | 0.57534564 |
| **ENSCAFG00000000231** | **B4GALT7** | **0.00054806** | **0.57534564** |
| ENSCAFG00000009270 | PEBP4 | 0.00069065 | 0.62184305 |
| ENSCAFG00000031187 | FGF12 | 0.00074199 | 0.63465944 |
| ENSCAFG00000005990 | C15H12ORF50 | 0.0007999 | 0.6516097 |
| ENSCAFG00000029971 | OTUD6B | 0.0009485 | 0.70701452 |
| ENSCAFG00000031487 | N/A | 0.00095772 | 0.70701452 |
| ENSCAFG00000018673 | SMARCA1 | 0.00102471 | 0.70701452 |
| ENSCAFG00000001299 | ZC3H3 | 0.00103322 | 0.70701452 |

Note - Q-values (33) were determined after correction for multiple hypothesis-testing of two foreground branches (bush dog and maned wolf) and 17,181 genes. The top 20 genes are shown. The gene associated with limb elongation is shown in bold.

**Table S7.** Candidate genes containing sites with signals of positive selection in the maned wolf, as identified by the branch-site test in PAML 4.8 (18).

| Ensmebl_ID | GeneSymbol | Pvalue | qvalue |
|---|---|---|---|
| ENSCAFG00000004101 | BICRA | 2.07E-08 | 0.00035604 |
| ENSCAFG00000010121 | CD81 | 4.43E-05 | 0.239424 |
| ENSCAFG00000005853 | PPP1R27 | 6.87E-05 | 0.239424 |
| ENSCAFG00000018235 | N/A | 6.96E-05 | 0.239424 |
| ENSCAFG00000019084 | NDC1 | 0.00010119 | 0.290078 |
| ENSCAFG00000001554 | BOP1 | 0.00014016 | 0.33940932 |
| ENSCAFG00000006785 | N/A | 0.00017288 | 0.33940932 |
| ENSCAFG00000017805 | REC114 | 0.00018369 | 0.33940932 |
| ENSCAFG00000016111 | FOXK1 | 0.00019733 | 0.33940932 |
| ENSCAFG00000011857 | SLAMF9 | 0.0002204 | 0.34462389 |
| ENSCAFG00000001372 | SCRIB | 0.0004468 | 0.57128844 |
| ENSCAFG00000009628 | GOLT1A | 0.00055128 | 0.57128844 |
| ENSCAFG00000030346 | N/A | 0.0005755 | 0.57128844 |
| ENSCAFG00000005759 | NPLOC4 | 0.00061634 | 0.57128844 |
| ENSCAFG00000031920 | N/A | 0.00064069 | 0.57128844 |
| ENSCAFG00000024210 | N/A | 0.00066799 | 0.57128844 |
| ENSCAFG00000010729 | MRGPRF | 0.00067789 | 0.57128844 |
| ENSCAFG00000001416 | GLDC | 0.00071138 | 0.57128844 |
| ENSCAFG00000010839 | LYPLAL1 | 0.00071388 | 0.57128844 |
| ENSCAFG00000028856 | N/A | 0.00077641 | 0.57128844 |

Note – Q-values (33) were determined after correction for multiple hypothesis-testing of two foreground branches (bush dog and maned wolf) and 17,181 genes. The top 20 genes are shown.

**Table S8.** Polygenic signals of selection in biological pathways in the maned wolf.

| setID | setSize | setScore | SetPvalue | SetQvalue | setName |
|---|---|---|---|---|---|
| **19** | **20** | **10.0479332** | **0.000497** | **0.01060714** | **Butanoate metabolism** |
| 27 | 14 | 4.6603052 | 0.09393177 | 0.71557709 | regulation of insulin secretion |
| 73 | 118 | 29.3100398 | 0.0962984 | 0.71557709 | spermatogenesis |
| 39 | 39 | 10.9415378 | 0.09708172 | 0.71557709 | Bile secretion |
| 34 | 19 | 5.86964581 | 0.1050979 | 0.71557709 | Proximal tubule bicarbonate reclamation |
| 42 | 30 | 8.25221796 | 0.14157146 | 0.76062436 | Fat digestion and absorption |
| 63 | 16 | 4.64562399 | 0.16719443 | 0.76062436 | sensory perception of bitter taste |
| 6 | 19 | 5.28137777 | 0.18489384 | 0.76062436 | Inositol phosphate metabolism, |
| 24 | 22 | 5.8305167 | 0.21159295 | 0.77857962 | Galactose metabolism |
| 14 | 10 | 2.70179973 | 0.27463627 | 0.8902873 | Pentose phosphate pathway |
| 36 | 34 | 8.0539239 | 0.30118494 | 0.8902873 | Salivary secretion |
| 17 | 19 | 4.46382865 | 0.34743263 | 0.8902873 | Propanoate metabolism |
| 18 | 10 | 2.33599555 | 0.36848158 | 0.8902873 | Pyruvate metabolism |
| 31 | 30 | 6.32340062 | 0.46627669 | 0.8902873 | Vasopressin-regulated water reabsorption |
| 16 | 10 | 2.02903521 | 0.47887606 | 0.8902873 | Pentose phosphate pathway |
| 11 | 19 | 3.95067003 | 0.48037598 | 0.8902873 | Citrate cycle (TCA cycle) |
| 41 | 50 | 10.4343825 | 0.49042548 | 0.8902873 | Protein digestion and absorption |
| 9 | 28 | 5.79945465 | 0.49242538 | 0.8902873 | Amino sugar and nucleotide sugar metabolism |
| 23 | 21 | 4.26092895 | 0.50514949 | 0.8902873 | Valine, leucine and isoleucine degradation |
| 8 | 51 | 10.5694061 | 0.51124888 | 0.8902873 | Phosphatidylinositol signaling system |
| 37 | 21 | 4.09006386 | 0.55224478 | 0.90233566 | Gastric acid secretion |
| 44 | 23 | 4.21779575 | 0.61633837 | 0.90233566 | Mineral absorption |
| 10 | 10 | 1.61389951 | 0.63333667 | 0.90233566 | Fructose and mannose metabolism |
| 26 | 53 | 10.0150271 | 0.66423358 | 0.90233566 | Vascular smooth muscle contraction |
| 32 | 15 | 2.40000638 | 0.68293171 | 0.90233566 | Aldosterone-regulated sodium reabsorption |
| 25 | 21 | 3.22233702 | 0.77252275 | 0.90233566 | Fatty acid metabolism |
| 3 | 52 | 9.03726468 | 0.77692231 | 0.90233566 | Adipocytokine signaling pathway |
| 20 | 42 | 6.94746229 | 0.7960204 | 0.90233566 | ErbB signaling pathway |
| 38 | 14 | 1.86028447 | 0.79612039 | 0.90233566 | Pancreatic secretion |
| 22 | 83 | 14.5817489 | 0.82381762 | 0.90233566 | mTOR signaling pathway |
| 1 | 32 | 4.89266487 | 0.82881712 | 0.90233566 | Insulin signaling pathway |
| 43 | 15 | 1.91973312 | 0.82891711 | 0.90233566 | Vitamin digestion and absorption |
| 33 | 20 | 2.55019202 | 0.86351365 | 0.90233566 | Endocrine and other factor-regulated calcium reabsorption |
| 2 | 14 | 1.56728725 | 0.86671333 | 0.90233566 | Glycolysis / Gluconeogenesis |
| 35 | 10 | 0.90679163 | 0.87051295 | 0.90233566 | Collecting duct acid secretion |
| 45 | 16 | 1.72343607 | 0.89881012 | 0.90629679 | Melanogenesis |

Note - For each pathway and the branch of the maned wolf, the ΔlnL4 values of the genes in different sets were calculated (see Supplementary Methods). The significance of each score was compared against a null distribution of random gene sets of the same size. The most significant pathway in the maned wolf was Butanoate metabolism (bold), which is related to energy intake from fruit fiber.

**Table S9** Polygenic signals of selection in biological pathways in the bush dog.

| setID | setSize | setScore | SetPvalue | SetQvalue | setName |
|---|---|---|---|---|---|
| 70 | 15 | 7.7269093 | 0.004717 | 0.15789448 | sensory perception of bitter taste |
| 11 | 20 | 7.72151245 | 0.04229965 | 0.48412229 | cholesterol homeostasis |
| 23 | 11 | 4.37578306 | 0.0862819 | 0.51508114 | glutamine metabolic process |
| 4 | 70 | 19.8530302 | 0.13384665 | 0.51508114 | lipid transport |
| 5 | 24 | 7.43698281 | 0.16577086 | 0.51508114 | unsaturated fatty acid metabolic process |
| 52 | 20 | 4.91918154 | 0.43417829 | 0.84746342 | positive regulation of lipid biosynthetic process |
| 56 | 11 | 2.65399489 | 0.4579271 | 0.84746342 | sensory perception of taste |
| 3 | 41 | 9.62180346 | 0.52794721 | 0.84981589 | cellular lipid catabolic process |
| 6 | 19 | 2.96092298 | 0.84091591 | 0.99339772 | regulation of lipid biosynthetic process |
| 32 | 15 | 2.03512374 | 0.87091291 | 0.99339772 | regulation of the fatty acid metabolic process |
| 1 | 22 | 2.76604225 | 0.94650535 | 0.99339772 | regulation of lipid metabolic process |
| 34 | 19 | 1.96879644 | 0.96350365 | 0.99339772 | positive regulation of lipid metabolic process |
| 2 | 30 | 2.84398229 | 0.99440056 | 0.99339772 | fatty acid metabolic process |

Note - For each pathway and the branch of the bush dog, the ΔlnL4 values of the genes in different sets were calculated (see Supplementary Methods). The significance of this score was compared against a null distribution of random gene sets of the same size.

**Table S10.** Polygenic signals of selection in the biological pathways of different canid species.

| Genome | setID | setSize | setScore | setPvalue | setQvalue | setName |
|---|---|---|---|---|---|---|
| African wild dog | 1819 | 371 | 131.398984 | 0.000423 | 0.31873598 | Olfactory transduction |
| African wild dog | 1009 | 78 | 31.7263022 | 0.00783436 | 0.99706243 | Class I MHC mediated antigen processing & presentation |
| African wild dog | 31 | 17 | 9.60770042 | 0.01201426 | 0.99706243 | Butanoate metabolism |
| African wild dog | 1393 | 13 | 7.92041778 | 0.01287945 | 0.99706243 | Pyrimidine metabolism |
| African wild dog | 654 | 16 | 9.0217327 | 0.01419718 | 0.99706243 | Endosomal Sorting Complex Required For Transport (ESCRT) |
| Dhole | 32691 | 375 | 87.8833681 | 0.000493 | 0.9907233 | Olfactory transduction |
| Dhole | 24494 | 10 | 6.04757047 | 0.00109 | 0.99436894 | negative regulation of innate immune response |
| Dhole | 2774 | 26 | 9.6684108 | 0.0052642 | 0.99436894 | Autophagy - other |
| Dhole | 19037 | 15 | 6.5676932 | 0.00614999 | 0.99436894 | aminoglycan catabolic process |
| Dhole | 3443 | 11 | 4.896259 | 0.01316407 | 0.99436894 | Selenocompound metabolism |
| Gray wolf | 1716 | 75 | 12.2017602 | 0.000751 | 0.45658585 | ECM-receptor interaction |
| Gray wolf | 1868 | 41 | 8.09818742 | 0.000984 | 0.45658585 | Type II diabetes mellitus |
| Gray wolf | 666 | 42 | 6.90991326 | 0.00725796 | 0.89423671 | Golgi Associated Vesicle Biogenesis |
| Gray wolf | 1113 | 18 | 3.90839539 | 0.00925635 | 0.89423671 | DNA strand elongation |
| Gray wolf | 1758 | 28 | 5.07223123 | 0.01059789 | 0.89423671 | Mucin type O-glycan biosynthesis |

Note - We calculated the ΔlnL4 values of the genes for each pathway and the branch of the coyote, gray wolf, dhole, and African wild dog (see Supplementary Methods). The significance of this score was compared against a null distribution of random gene sets of the same size.

**Table S11.** Scores of private alleles (SinglBySeg) found in 1 kb windows flanking 39,704 transcripts in the bush dog.

| chromo | Start | End | SinglBySeg | Sites Passing | EnsemblID | Gene |
|---|---|---|---|---|---|---|
| chr14 | 7814773 | 7815773 | 3.611111 | 661 | ENSCAFG00000001585 | FLNC |
| chr15 | 41275699 | 41276699 | 2.6 | 959 | ENSCAFG00000007304 | **IGF1** |
| chr31 | 34355444 | 34356444 | 2.6 | 604 | ENSCAFG00000029475 | **B3GALT5** |
| chr33 | 18571299 | 18572299 | 2.51875 | 755 | ENSCAFG00000010823 | ZBTB20 |
| chr07 | 28137504 | 28138504 | 2.47619 | 802 | ENSCAFG00000015002 | PRRX1 |
| chr03 | 19850617 | 19851617 | 2.363636 | 965 | ENSCAFG00000008302 | MEF2C |
| chr16 | 27257586 | 27258586 | 2.26087 | 501 | ENSCAFG00000006099 | DDHD2 |
| chr32 | 24857235 | 24858235 | 2.166667 | 742 | ENSCAFG00000010855 | TACR3 |
| chr06 | 17449879 | 17450879 | 2.166667 | 664 | ENSCAFG00000016640 | ENSCAFG-00000016640 |
| chr32 | 24857235 | 24858235 | 2.166667 | 742 | ENSCAFG00000010855 | TACR3 |
| chr11 | 21219637 | 21220637 | 1.925926 | 565 | ENSCAFG00000000930 | AFF4 |
| chr26 | 30082470 | 30083470 | 1.925926 | 579 | ENSCAFG00000014833 | DGCR2 |
| chr15 | 18315887 | 18316887 | 1.902439 | 956 | ENSCAFG00000005511 | RPGRIP1 |
| chr15 | 18181960 | 18182960 | 1.882759 | 740 | ENSCAFG00000005493 | ZNF219 |
| chr11 | 52518271 | 52519271 | 1.833333 | 546 | ENSCAFG00000002281 | RECK |
| chr18 | 46299919 | 46300919 | 1.813953 | 989 | ENSCAFG00000010090 | IGF2 |
| chr20 | 50487161 | 50488161 | 1.813953 | 574 | ENSCAFG00000017651 | DNM2 |

Note – The ‘SinglBySeg’ column represents the proportion of private alleles in bush dogs divided by the average of variable sites among other canids (see Supplementary Methods for details). The top 20 Candidate genes are shown. The genes related to bone elongation are shown in red.

**Table S12.** Scores of private alleles (SinglBySeg) found in 1kb windows flanking 39,704 transcripts in the maned wolf.

| chromo | Start | End | SinglBySeg | Sites Passing | EnsemblID | Gene |
|---|---|---|---|---|---|---|
| chr20 | 8722345 | 8723345 | 3.25 | 998 | ENSCAFG00000005486 | SETD5 |
| chr12 | 23936471 | 23937471 | 2.666667 | 750 | ENSCAFG00000002413 | DST |
| chr03 | 64867819 | 64868819 | 2.409756 | 532 | ENSCAFG00000015345 | CC2D2A |
| chr14 | 40348900 | 40349900 | 2.166667 | 616 | ENSCAFG00000002963 | HOXA10 |
| chr11 | 3505713 | 3506713 | 2 | 767 | ENSCAFG00000000231 | **B4GALT7** |
| chr02 | 21120659 | 21121659 | 1.890909 | 579 | ENSCAFG00000004656 | ITGA8 |
| chr17 | 39418364 | 39419364 | 1.772727 | 949 | ENSCAFG00000007658 | SFTPB |
| chr30 | 38284938 | 38285938 | 1.733333 | 584 | ENSCAFG00000018010 | SIN3A |
| chr12 | 511945 | 512945 | 1.666667 | 584 | ENSCAFG00000000443 | ENSCAFG-00000000443 |
| chr23 | 1562844 | 1563844 | 1.659574 | 597 | ENSCAFG00000004389 | ABHD12 |
| chr34 | 33807613 | 33808613 | 1.63913 | 961 | ENSCAFG00000014621 | MECOM |
| chr04 | 71697274 | 71698274 | 1.625 | 761 | ENSCAFG00000018695 | NIPBL |
| chr01 | 116756289 | 116757289 | 1.591837 | 709 | ENSCAFG00000006697 | ENSCAFG-00000006697 |
| chr10 | 308502 | 309502 | 1.5 | 986 | ENSCAFG00000000080 | PMEL |
| chr26 | 22725593 | 22726593 | 1.493243 | 654 | ENSCAFG00000012243 | NEFH |
| chr32 | 12313144 | 12314144 | 1.474627 | 950 | ENSCAFG00000009840 | FAM13A |
| chr11 | 21218186 | 21219186 | 1.4625 | 828 | ENSCAFG00000000930 | AFF4 |
| chr12 | 2954835 | 2955835 | 1.460674 | 907 | ENSCAFG00000001022 | BAK1 |

Note – The 'SinglBySeg' column represents the proportion of private alleles in the maned wolf divided by the mean of variable sites among other canids (see Supplementary Methods for details). The top 20 candidate genes are shown. The gene related to bone elongation is shown in red.

**Table S13**. List of genes enriched for private alleles found in 1kb windows flanking 39,704 transcripts in different canid species

| Species | Limb-related genes | chondrogenesis-related genes |
|---|---|---|
| Bush dog | FLNC,**IGF1**,**B3GALT5**,IGF1,B3GALT5,ZBTB20,PRRX1,MEF2C,DDHD2,TACR3,TACR3,AFF4,DGCR2,AFF4,DGCR2,RPGRIP1,ZNF219 | **IGF1** **B3GALT5** |
| Maned wolf | SETD5,DST,CC2D2A,HOXA10,**B4GALT7**,ITGA8,SFTPB,SIN3A | **B4GALT7** |
| Coyote | KLC2,GLI1,BRF1,RBPJ,CITED1,UBE2T,CACNA1C,KCNA1,BRAF,DTNBP1 | NA |
| Golden Wolf | PORCN,CLCF1,CITED1,DTNBP1,GLRA1,MYCBP | NA |
| Side-striped Jackal | TGFB2,PEX16,POMT2,WNT10A,CITED2,IRX5 | NA |
| Black-backed Jackal | MEIS2,DGCR2,BMP5,NR2E1,ADNP,UBA1 | NA |
| Wild dog | ABHD12,COL9A3,LRBA,COL1A1,ERLIN1 | NA |
| Culpeo Fox | EGR2,ADNP,GALNS,RERE,SMC1A,RIN2 | NA |
| Ethiopian_wolf | SP9,LTBP4,KLC2,LZTR1,GJA1,KRT10 | NA |
| Dhole | EGR2,ADNP | NA |
| Gray Wolf | NA | NA |

Note- We chose species in different clades across the species tree (Figure S1). Only genes with empirical p-values less than 0.01 are shown. Specifically, we filtered windows with less than 500 sites with data and then chose the value of P/S that indicated the top 1% of the remaining number of windows. Notice that bush dog has an overrepresentation of limb-related genes. Only the maned wolf and bush dog have chondrogenesis-related genes on their lists.

**Table S14**. Assembly Statistics for the Maned wolf and Bush Dog

| Maned Wolf | | | Bush Dog | |
|---|---|---|---|---|
| | Contig | Scaffold | Contig | Scaffolds |
| L10 | 1025 | 104 | 1701 | 118 |
| L20 | 2615 | 254 | 4250 | 302 |
| L30 | 4692 | 447 | 7546 | 542 |
| L40 | 7320 | 685 | 11651 | 839 |
| L50 | 10606 | 972 | 16741 | 1209 |
| N10 | 172366 | 1771093 | 106282 | 1488311 |
| N20 | 126074 | 1363687 | 79028 | 1095955 |
| N30 | 98808 | 1078784 | 62799 | 880438 |
| N40 | 78896 | 889626 | 50429 | 706602 |
| N50 | 62925 | 739658 | 40867 | 571622 |
| gc content | 41.037 | 41.037 | 41.005 | 41.005 |
| longest | 550768 | 4378521 | 364261 | 4605694 |
| mean | 9176.621 | 13900.113 | 8092.413 | 13839.067 |
| median | 101.0 | 101.0 | 303.0 | 101.0 |
| Sequence count | 251901 | 167868 | 285210 | 169014 |
| shortest | 64 | 64 | 64 | 64 |
| Total bps | 2311600122 | 2333384174 | 2308037114 | 2338996162 |

**Table S15**. Repetitive Elements in the Maned Wolf and Bush Dog

| | Maned wolf | | | | Bush Dog | | |
|---|---|---|---|---|---|---|---|
| | Number of elements | Length occupied (bp) | Percentage of sequence (%) | | Number of elements | Length occupied (bp) | Percentage of sequence (%) |
| Total Interspersed Repeats | | 637544093 | 27.32 | | | 634623023 | 27.13 |
| **SINEs:** | 438268 | 64263306 | 2.75 | **SINEs:** | 436379 | 63949386 | 2.73 |
| Alu/B1 | 0 | 0 | 0.00 | Alu/B1 | 0 | 0 | 0.00 |
| MIRs | 431421 | 63438514 | 2.72 | MIRs | 429614 | 63133586 | 2.70 |
| **LINEs:** | 815839 | 406536273 | 17.42 | **LINEs:** | 823321 | 404783531 | 17.31 |
| LINE1 | 476673 | 317897232 | 13.62 | LINE1 | 485857 | 316779837 | 13.54 |
| LINE2 | 288327 | 77387282 | 3.32 | LINE2 | 286950 | 76862216 | 3.29 |
| L3/CR1 | 38050 | 8248159 | 0.35 | L3/CR1 | 37870 | 8191456 | 0.35 |
| RTE | 11563 | 2810021 | 0.12 | RTE | 11453 | 2759211 | 0.12 |
| **LTR elements:** | 274091 | 101391803 | 4.35 | **LTR elements:** | 273310 | 100858680 | 4.31 |
| ERVL | 83270 | 36841284 | 1.58 | ERVL | 82998 | 36643357 | 1.57 |
| ERVL-MaLRs | 139608 | 48113829 | 2.06 | ERVL-MaLRs | 139297 | 47960425 | 2.05 |
| ERV_classI | 27172 | 11117381 | 0.48 | ERV_classI | 27054 | 10949313 | 0.47 |
| ERV_classII | 0 | 0 | 0.00 | ERV_classII | 0 | 0 | 0.00 |
| **DNA elements:** | 319314 | 64797079 | 2.78 | **DNA elements:** | 318364 | 64472291 | 2.76 |
| hAT-Charlie | 183966 | 34757018 | 1.49 | hAT-Charlie | 183471 | 34592343 | 1.48 |
| TcMar-Tigger | 51268 | 13708011 | 0.59 | TcMar-Tigger | 51043 | 13641282 | 0.58 |
| Unclassified: | 3553 | 555632 | 0.02 | Unclassified: | 3556 | 559135 | 0.02 |

**Table S16.** Set of fossil calibration priors (minimum and maximum node ages) used for the MCMCTree analyses.

| Node on tree | Node | Node minimum age (Ma) | Node maximum age (Ma) | Evidence | Oldest fossil taxon | Prior model |
|---|---|---|---|---|---|---|
| **1** | Canini - Vulpini split | 9 | 11.9 | Tedford et al. 2009; Slater et al. 2012; Austin et al. 2013 | *Eucyon davisi* | Lognormal |
| **2** | TMRCA crown Cerdocyonina | 5 | 9 | Prevosti 2010; Slater et al. 2012; Austin et al. 2013 | Chrysocyon nearcticus | Lognormal |
| **3** | TMRCA crown *Lycalopex* | 2.588 | 3.6 | Prevosti 2010; 2018 (and references therin) | *Lycalopex cultridens* | Lognormal |
| **4** | *Lycalopex culpaeus* - *L. sechurae* split | 1.1 | 3 | Tedford et al. 2009; Slater et al. 2012 | *Canis edwardsii* | Lognormal |

Note – Average genomic divergence time estimates are shown in Figure S1 and Table S18.

**Table S17.** Generation time estimates for canids

| Common name | Species | Generation length/time (years) - Pacifici et al 2013 | Generation length/time (years) - IUCN Red List; other |
|---|---|---|---|
| Maned wolf | *Chrysocyon brachyurus* | 5.6 | 5 |
| Bush dog | *Speothos venaticus* | 4.3 | 4 |
| Crab-eating fox | *Cerdocyon thous* | 4.2 | |
| Small-eared dog | *Atelocynus microtus* | 3.9 | 4 |
| Hoary fox | *Lycalopex vetulus* | 4 | 3.5 |
| Pampas fox | *Lycalopex gymnocercus* | 4.7 | 3.5 |
| South American gray fox | *Lycalopex griseus* | 4.2 | 3.5 |
| Darwin's fox | *Lycalopex fulvipes* | 3.8 | 3.5 |
| Culpeo fox | *Lycalopex culpaeus* | 4.6 | 3.5 |
| Sechuran fox | *Lycalopex sechurae* | 4 | 3 |
| Coyote | *Canis latrans* | 7 | 4 |
| Gray wolf | *Canis lupus* | 7.9 | 4.2-4.7** |
| Black-backed jackal | *Lupullela mesomelas* | 5 | |
| Gray fox | *Urocyon cinereoargenteus* | 5.4 | 3 |

Note Definition used for generation length/time: "the average age of parents of the current cohort, reflecting the turnover rate of breeding individuals in a population". Estimates based on  Pacifici at al (23), IUCN Red List (71-80) and other (81).

**Table S18.** Divergence estimates for different internal nodes in the species tree of South American canids inferred by MCMCTree (82) and G-PhoCS (https://github.com/gphocs-dev/G-PhoCS).

| Node on the Tree | Clade corresponding to a specific node in the tree | Average times for common ancestry with MCMC | Species divergence times with G-PhoCs |
|---|---|---|---|
| N24 | Ancestral to canids in South America | 3.39 (1.53-4.47) | 3.51 (3.50-3.53) |
| N14 | Bush dog + Maned wolf | 3.05 (1.35-4.34) | 3.10 (3.05-3.15) |
| N23 | Short-eared dog + Crab-eating fox + *Lycalopex* | 2.61 (1.15-3.59) | 2.25 (2.23-2.27) |
| N15 | Short-eared dog + Crab-eating fox | 2.11 (0.92-3.06) | 1.87 (1.81-1.92) |
| N22 | *Lycalopex* | 1.98 (0.88-2.81)) | 1.40 (1.36-1.43) |
| N21 | Sechuran fox + Culpeo fox + Darwin's fox + SA gray fox + Pampas fox | 1.72 (0.77-2.48) | 1.02 (0.98-1.04) |
| N20 | Sechuran fox + Culpeo fox + Darwin's fox | 1.49 (0.66-2.18) | 1.01 (0.98-1.04) |
| N17 | SA gray fox + Pampas fox | 1.21 (0.52-1.89) | 1.01 (0.98-1.04) |
| N19 | Sechuran fox + Culpeo fox | 1.04 (0.44-1.61) | 0.81 (0.78-0.84) |

Note – The tree with internal nodes is shown in Figure 2 for Species divergence inferred by G-PhoCs and Figure S1 for Average times inferred by MCMC.

## References


1. A. V. Zimin *et al.*, The MaSuRCA genome assembler. *Bioinformatics* **29**, 2669-2677 (2013).

2. G. Marcais, J. A. Yorke, A. Zimin, QuorUM: An Error Corrector for Illumina Reads. *Plos One* **10** (2015).

3. J. R. Miller *et al.*, Aggressive assembly of pyrosequencing reads with mates. *Bioinformatics* **24**, 2818-2824 (2008).

4. M. Trizna (2020) assembly_stats 0.1.4. (Zenodo).

5. F. A. Simao, R. M. Waterhouse, P. Ioannidis, E. V. Kriventseva, E. M. Zdobnov, BUSCO: assessing genome assembly and annotation completeness with single-copy orthologs. *Bioinformatics* **31**, 3210-3212 (2015).

6. A. McKenna *et al.*, The Genome Analysis Toolkit: A MapReduce framework for analyzing next-generation DNA sequencing data. *Genome Research* **20**, 1297-1303 (2010).

7. G. A. Van der Auwera, B. D. O'Connor, *Genomics in the Cloud: Using Docker, GATK, and WDL in Terra* (O'Reilly Media, 2020).

8. K. Lindblad-Toh *et al.*, Genome sequence, comparative analysis and haplotype structure of the domestic dog. *Nature* **438**, 803-819 (2005).

9. H. Li, Aligning sequence reads, clone sequences and assembly contigs with BWA-MEM.

10. J. A. Robinson *et al.*, Genomic signatures of extensive inbreeding in Isle Royale wolves, a population on the threshold of extinction. *Science Advances* **5** (2019).

11. C. Zhang, M. Rabiee, E. Sayyari, S. Mirarab, ASTRAL-III: polynomial time species tree reconstruction from partially resolved gene trees. *Bmc Bioinformatics* **19** (2018).

12. A. R. Quinlan, I. M. Hall, BEDTools: a flexible suite of utilities for comparing genomic features. *Bioinformatics* **26**, 841-842 (2010).

13. A. Loytynoja, N. Goldman, Phylogeny-aware gap placement prevents errors in sequence alignment and evolutionary analysis. *Science* **320**, 1632-1635 (2008).

14. A. Loytynoja, N. Goldman, An algorithm for progressive multiple alignment of sequences with insertions. *Proceedings of the National Academy of Sciences of the United States of America* **102**, 10557-10562 (2005).

15. J. Castresana, Selection of conserved blocks from multiple alignments for their use in phylogenetic analysis. *Molecular Biology and Evolution* **17**, 540-552 (2000).

16. L. T. Nguyen, H. A. Schmidt, A. von Haeseler, B. Q. Minh, IQ-TREE: A Fast and Effective Stochastic Algorithm for Estimating Maximum-Likelihood Phylogenies. *Molecular Biology and Evolution* **32**, 268-274 (2015).

17. S. Kalyaanamoorthy, B. Q. Minh, T. K. F. Wong, A. von Haeseler, L. S. Jermiin, ModelFinder: fast model selection for accurate phylogenetic estimates. *Nature Methods* **14**, 587-+ (2017).

18. Z. H. Yang, PAML 4: Phylogenetic analysis by maximum likelihood. *Molecular Biology and Evolution* **24**, 1586-1591 (2007).

19. M. Hasegawa, H. Kishino, T. A. Yano, DATING OF THE HUMAN APE SPLITTING BY A MOLECULAR CLOCK OF MITOCHONDRIAL-DNA. *Journal of Molecular Evolution* **22**, 160-174 (1985).

20. N. J. Matzke (2013) BioGeoBEARS: biogeography with Bayesian (and likelihood) evolutionary analysis in R Scripts. (University of California, Berkeley, Berkeley, CA).

21. D. E. Chavez *et al.*, Comparative genomics provides new insights into the remarkable adaptations of the African wild dog (Lycaon pictus). *Scientific Reports* **9** (2019).

22. P. Skoglund, E. Ersmark, E. Palkopoulou, L. Dalen, Ancient Wolf Genome Reveals an Early Divergence of Domestic Dog Ancestors and Admixture into High-Latitude Breeds. *Current Biology* **25**, 1515-1519 (2015).

23. M. Pacifici *et al.*, Generation length for mammals. *Nature Conservation-Bulgaria*, 87-94 (2013).

24. S. Purcell *et al.*, PLINK: A tool set for whole-genome association and population-based linkage analyses. *American Journal of Human Genetics* **81**, 559-575 (2007).

25. S. Schiffels, R. Durbin, Inferring human population size and separation history from multiple genome sequences. *Nature Genetics* **46**, 919-925 (2014).

26. A. C. Beichman *et al.*, Aquatic Adaptation and Depleted Diversity: A Deep Dive into the Genomes of the Sea Otter and Giant Otter. *Molecular Biology and Evolution* **36**, 2631-2655 (2019).

27. B. M. vonHoldt *et al.*, Whole-genome sequence analysis shows that two endemic species of North American wolf are admixtures of the coyote and gray wolf. *Science Advances* **2** (2016).

28. P. U. Clark *et al.*, The Last Glacial Maximum. *Science* **325**, 710-714 (2009).

29. A. E. Webb, T. A. Walsh, M. J. O'Connell, VESPA: Very large-scale Evolutionary and Selective Pressure Analyses. *PeerJ Computer Science* **3**, 1-16 (2017).

30. P. W. Harrison, G. E. Jordan, S. H. Montgomery, SWAMP: Sliding Window Alignment Masker for PAML. *Evolutionary Bioinformatics* **10** (2014).

31. A. Venkat, M. W. Hahn, J. W. Thornton, Multinucleotide mutations cause false inferences of lineage-specific positive selection. *Nature Ecology & Evolution* **2**, 1280-1288 (2018).

32. J. Z. Zhang, R. Nielsen, Z. H. Yang, Evaluation of an improved branch-site likelihood method for detecting positive selection at the molecular level. *Molecular Biology and Evolution* **22**, 2472-2479 (2005).

33. J. D. Storey, A. J. Bass, A. Dabney, D. Robinson (2021) qvalue: Q-value estimation for false discovery rate control. (R package version 2.24.0), p http://github.com/jdstorey/qvalue .

34. S. F. Altschul, W. Gish, W. Miller, E. W. Myers, D. J. Lipman, BASIC LOCAL ALIGNMENT SEARCH TOOL. *Journal of Molecular Biology* **215**, 403-410 (1990).

35. J. T. Daub, S. Moretti, Davydov, II, L. Excoffier, M. Robinson-Rechavi, Detection of Pathways Affected by Positive Selection in Primate Lineages Ancestral to Humans. *Molecular Biology and Evolution* **34**, 1391-1402 (2017).

36. W. McLaren *et al.*, The Ensembl Variant Effect Predictor. *Genome Biology* **17** (2016).

37. J. A. Robinson *et al.*, Genomic Flatlining in the Endangered Island Fox. *Current Biology* **26**, 1183-1189 (2016).

38. C. D. Marsden *et al.*, Bottlenecks and selective sweeps during domestication have increased deleterious genetic variation in dogs. *Proceedings of the National Academy of Sciences of the United States of America* **113**, 152-157 (2016).

39. K. E. Lohmueller *et al.*, Proportionally more deleterious genetic variation in European than in African populations. *Nature* **451**, 994-U995 (2008).

40. K. Angelis, M. Dos Reis, The impact of ancestral population size and incomplete lineage sorting on Bayesian estimation of species divergence times. *Current Zoology* **61**, 874-885 (2015).

41. M. Dos Reis, Z. H. Yang, The unbearable uncertainty of Bayesian divergence time estimation. *Journal of Systematics and Evolution* **51**, 30-43 (2013).

42. G. J. Slater *et al.*, Evolutionary history of the Falklands wolf. *Current Biology* **19**, R937-R938 (2009).

43. F. A. Perini, C. A. M. Russo, C. G. Schrago, The evolution of South American endemic canids: a history of rapid diversification and morphological parallelism. *Journal of Evolutionary Biology* **23**, 311-322 (2010).

44. L. Tchaicka *et al.*, Molecular assessment of the phylogeny and biogeography of a recently diversified endemic group of South American canids (Mammalia: Carnivora: Canidae). *Genetics and Molecular Biology* **39**, 442-451 (2016).

45. R. H. Tedford, X. Wang, B. E. Taylor, Phylogenetic systematics of the North American fossil Caninae (Carnivora: Canidae). *Bulletin of the American Museum of Natural History* **325**, 1-218 (2009).

46. V. Torres-Roldán, I. Ferrusquía-Villafranca (1981) *Cerdocyon sp. nov*. A. (Mammalia, Carnivora) en México y su significacién evolutiva y zoogeográfica en relación a los cánidos sudamericanos. in *II Congresso Latino-Americano Paleontologia* (Porto Alegre), pp 709-719.

47. A. Berta, *Origin, diversification, and zoogeography of the South American Canidae* (Fieldiana. Zoology[FIELDIANA ZOOL.], 1987).

48. A. O'Dea *et al.*, Formation of the Isthmus of Panama. *Science Advances* **2** (2016).

49. C. D. Bacon *et al.*, Biological evidence supports an early and complex emergence of the Isthmus of Panama (vol 112, pg 6110, 2015). *Proceedings of the National Academy of Sciences of the United States of America* **112**, E3631-E3631 (2015).

50. A. Berta, The Pleistocene Bush Dog *Speothos pacivorus* (Canidae) from the Lagoa Santa Caves, Brazil. *Journal of Mammalogy* **65**, 549-559 (1984).

51. R. H. Tedford, Z. Qiu, Pliocene *Nyctereutes* (Carnivora: Canidae) from Yushe, Shanxi, with comments on Chinese fossil Racoon-dogs.

52. X. Wang, R. H. Tedford, M. AntÓN, *Dogs: Their Fossil Relatives and Evolutionary History* (Columbia University Press, 2008).

53. F. J. Prevosti, Phylogeny of the large extinct South American Canids (Mammalia, Carnivora, Canidae) using a "total evidence" approach. *Cladistics* **26**, 456-481 (2010).

54. L. Nahuelhual *et al.*, Valuing ecosystem services of Chilean temperate rainforests. *Environment, Development and Sustainability* **9**, 481-499 (2007).

55. J. Kaiser, F. Lamy, H. W. Arz, D. Hebbeln, Dynamics of the millennial-scale sea surface temperature and Patagonian Ice Sheet fluctuations in southern Chile during the last 70kyr (ODP Site 1233). *Quaternary International* **161**, 77-89 (2007).

56. C. Villagran, Late Quaternary Vegetation of Southern Isla Grande de Chiloé, Chile. *Quaternary Research* **29**, 294-306 (1988).

57. S. Otavo, C. Echeverria, Fragmentación progresiva y pérdida de hábitat de bosques naturales en uno de los hotspot mundiales de biodiversidad. *Revista Mexicana de Biodiversidad* **88** (2017).

58. A. A. Farias *et al.*, A new population of Darwin's fox (*Lycalopex fulvipes*) in the Valdivian Coastal Range. *Revista Chilena De Historia Natural* **87** (2014).

59. C. J. Yahnke *et al.*, Darwin's fox: A distinct endangered species in a vanishing habitat. *Conservation Biology* **10**, 366-375 (1996).

60. J. E. Jiménez, E. McMahon, "*Pseudalopex fulvipes*" in Canids: Foxes, wolves, jackals and dogs, C. Sillero-Zubiri, M. Hoffmann, D. W. Macdonald, Eds. (Status survey and conservation action plan, IUCN/SSC Canid Specialist Group, Gland, Switzerland and Cambridge, UK, 2015).

61. E. Silva-Rodríguez *et al.* (2016) *Lycalopex fulvipes* (errata version published in 2016). The IUCN Red List of Threatened Species 2016: e.T41586A107263066. (https://dx.doi.org/10.2305/IUCN.UK.2016-1.RLTS.T41586A85370871.en . ).

62. A. Miranda, A. Altamirano, L. Cayuela, F. Pincheira, A. Lara, Different times, same story: Native forest loss and landscape homogenization in three physiographical areas of south-central of Chile. *Applied Geography* **60**, 20-28 (2015).

63. A. R. Perri *et al.*, Dire wolves were the last of an ancient New World canid lineage. *Nature* **591**, 87-+ (2021).

64. K. P. Koepfli *et al.*, Genome-wide Evidence Reveals that African and Eurasian Golden Jackals Are Distinct Species. *Current Biology* **25**, 2158-2165 (2015).

65. A. Auton *et al.*, Genetic Recombination Is Targeted towards Gene Promoter Regions in Dogs. *Plos Genetics* **9** (2013).

66. S. Gopalakrishnan *et al.*, Interspecific Gene Flow Shaped the Evolution of the Genus Canis. *Current Biology* **28**, 3441-3449 (2018).

67. A. H. Freedman *et al.*, Genome Sequencing Highlights the Dynamic Early History of Dogs. *Plos Genetics* **10** (2014).

68. Z. X. Fan *et al.*, Worldwide patterns of genomic variation and admixture in gray wolves. *Genome Research* **26**, 163-173 (2016).

69. A. V. Kukekova *et al.*, Red fox genome assembly identifies genomic regions associated with tame and aggressive behaviours. *Nature Ecology & Evolution* **2**, 1479-1491 (2018).

70. K. Okonechnikov, A. Conesa, F. Garcia-Alcalde, Qualimap 2: advanced multi-sample quality control for high-throughput sequencing data. *Bioinformatics* **32**, 292-294 (2016).

71. R. C. Paula, K. DeMatteo, Chrysocyon brachyurus (errata version published in 2016). The IUCN Red List of Threatened Species 2015: e.T4819A88135664. https://dx.doi.org/10.2305.

72. K. DeMatteo, F. Michalski , M. R. P. Leite-Pitman, Speothos venaticus. The IUCN Red List of Threatened Species 2011: e.T20468A9203243. https://dx.doi.org/10.2305/IUCN.UK.2011-2.RLTS.T20468A9203243.en.

73. M. R. P. Leite-Pitman, R. S. R. Williams, Atelocynus microtis. The IUCN Red List of Threatened Species 2011: e.T6924A12814890. https://dx.doi.org/10.2305/IUCN.UK.2011-2.RLTS.T6924A12814890.en.

74. F. G. Lemos, F. C. Azevedo, R. C. Paula, J. C. Dalponte (2020) Lycalopex vetulus. The IUCN Red List of Threatened Species 2020: e.T6926A87695615. https://dx.doi.org/10.2305/IUCN.UK.2020-2.RLTS.T6926A87695615.en.

75. M. Lucherini, Lycalopex gymnocercus. The IUCN Red List of Threatened Species 2016: e.T6928A85371194. https://dx.doi.org/10.2305/IUCN.UK.2016-1.RLTS.T6928A85371194.en.

76. M. Lucherini, Lycalopex griseus (errata version published in 2017). The IUCN Red List of Threatened Species 2016: e.T6927A111975602. https://dx.doi.org/10.2305/IUCN.UK.2016-1.RLTS.T6927A86440397.en.

77. E. Silva-Rodríguez *et al.*, Lycalopex fulvipes (errata version published in 2016). The IUCN Red List of Threatened Species 2016: e.T41586A107263066. https://dx.doi.org/10.2305/IUCN.UK.2016-1.RLTS.T41586A85370871.en.

78. D. Cossios, Lycalopex sechurae. The IUCN Red List of Threatened Species 2017: e.T6925A86074993. https://dx.doi.org/10.2305/IUCN.UK.2017-2.RLTS.T6925A86074993.en.

79. R. Kays, Canis latrans (errata version published in 2020). The IUCN Red List of Threatened Species 2018: e.T3745A163508579. https://dx.doi.org/10.2305/IUCN.UK.2018-2.RLTS.T3745A163508579.en.

80. G. Roemer, B. Cypher, R. List, Urocyon cinereoargenteus. The IUCN Red List of Threatened Species 2016: e.T22780A46178068. https://dx.doi.org/10.2305/IUCN.UK.2016-1.RLTS.T22780A46178068.en.

81. L. D. Mech, S. M. Barber-Meyer, Use of Erroneous Wolf Generation time in Assessments of Domestic Dog and Human Evolution. E-letter in Genomic and archaeological evidence suggest a dual origin of domestic dogs. Frantz, L. A., et al. Science 352(6290):1228-1231.

82. Z. H. Yang, PAML: a program package for phylogenetic analysis by maximum likelihood. *Computer Applications in the Biosciences* **13**, 555-556 (1997).
